# Towards the extraction of the crystal cell parameters from pair distribution function profiles

**DOI:** 10.1107/S2052252523006887

**Published:** 2023-09-01

**Authors:** Pietro Guccione, Domenico Diacono, Stefano Toso, Rocco Caliandro

**Affiliations:** a Dipartimento di Ingegneria Elettrica e dell’Informazione, Politecnico di Bari, via Orabona 4, Bari 70125, Italy; b INFN Sezione di Bari, via Orabona 4, Bari 70125, Italy; c Italian Institute of Technology, via Morego 30, Genoa 16163, Italy; d Institute of Crystallography, National Research Council of Italy, via Amendola 122/o, Bari 70126, Italy; Alfred University, USA

**Keywords:** crystal cell parameters, crystal lattices, pair distribution functions, nanocrystals, machine learning, multivariate analysis, vector superpositions

## Abstract

The approach based on atomic pair distribution function (PDF) has revolutionized structural investigations by X-ray/electron diffraction of nano or quasi-amorphous materials, opening up the possibility of exploring short-range order. However, the *ab initio* crystal structural solution by the PDF is far from being achieved due to the difficulty in determining the crystallographic properties of the unit cell. A method for estimating the crystal cell parameters directly from a PDF profile is presented, which is composed of two steps: first, the type of crystal cell is inferred using machine-learning approaches applied to the PDF profile; second, the crystal cell parameters are extracted by means of multivariate analysis combined with vector superposition techniques. The procedure has been validated on a large number of PDF profiles calculated from known crystal structures and on a small number of measured PDF profiles. The lattice determination step has been benchmarked by a comprehensive exploration of different classifiers and different input data. The highest performance is obtained using the *k*-nearest neighbours classifier applied to whole PDF profiles. Descriptors calculated from the PDF profiles by recurrence quantitative analysis produce results that can be interpreted in terms of PDF properties, and the significance of each descriptor in determining the prediction is evaluated. The cell parameter extraction step depends on the cell metric rather than its type. Monometric, dimetric and trimetric cells have top-1 estimates that are correct 40, 20 and 5% of the time, respectively. Promising results were obtained when analysing real nanocrystals, where unit cells close to the true ones are found within the top-1 ranked solution in the case of monometric cells and within the top-6 ranked solutions in the case of dimetric cells, even in the presence of a crystalline impurity with a weight fraction up to 40%.

## Abstract 2

A method to estimate crystal cell parameters directly from a pair distribution function profile, which makes use of machine-learning approaches combined with multivariate analysis and vector superposition techniques, is presented.

## 1. Introduction

X-ray or electron powder diffraction allows us to infer structural information at atomic resolution for materials or organic molecules for which large crystals (less than a few micrometres) are not available (Billinge, 2019 ▸; Junior *et al.*, 2021 ▸). Although power diffraction is less informative than single-crystal diffraction due to the collapse of the three-dimensional reciprocal lattice in a unidimensional diffraction pattern, it is much faster, and complete datasets can be collected in a few seconds. In addition, the advent of new-generation X-ray sources and faster data acquisition technologies has opened the possibility of monitoring structural features of dynamic processes such as phase transitions (Caliandro *et al.*, 2019 ▸; Pang *et al.*, 2022 ▸), electrochemical (Cañas *et al.*, 2017 ▸) or mechanochemical (Katsenis *et al.*, 2015 ▸) reactions, and crystallization (Davey *et al.*, 2002 ▸) by means of *in situ* or even *operando* experiments.

Besides the complexity of the investigated processes, the nature of the samples analysed has also become a challenge. Complex materials such as quantum dots (Uragami *et al.*, 2002 ▸), nanoclusters (Zhang *et al.*, 2022 ▸), pharmaceuticals (Garcia-Bennett *et al.*, 2018 ▸), metal–organic frameworks (Koschnick *et al.*, 2021 ▸) and quasi-crystals (Fan *et al.*, 2006 ▸) suffer from lattice defects, surface effects, structural disorder and low crystallinity, which disrupt long-range order typical of crystalline compounds. The local structure of such samples can be investigated by the pair distribution function (PDF), which is a one-dimensional real space function that describes how the atomic density varies over distance. In particular, the reduced PDF *G*(*r*) is a measure of the probability of finding an atom pair separated by the interatomic distance *r*, weighted by the scattering factors of the atoms in that pair (Neder & Proffen, 2008 ▸; Egami & Billinge, 2012 ▸). It is calculated by considering the X-ray/electron scattered intensity along diffraction maxima (the so-called Bragg peaks), as well as that arising from diffuse scattering. Such a total scattering technique has access to short-range order and is able to reveal structural information not only of solid samples but also of colloidal dispersions and even solutions. It is frequently used for qualitative and quantitative phase analysis (Zea-Garcia *et al.*, 2019 ▸); determination of the average domain size (Kodama *et al.*, 2006 ▸) and the amorphous content (Peterson *et al.*, 2013 ▸); or to disclose local structural features of inorganic materials (Colella *et al.*, 2018 ▸), liquid and glasses (Juhás *et al.*, 2010 ▸).

The work towards obtaining useful information for structural resolution from the PDF was started by the development of algorithms to extract the peak position (Granlund *et al.*, 2015 ▸) and the distance list (Gu *et al.*, 2019 ▸) from PDF profiles. They automatically recover the peak position with no *a priori* structural information, taking into account aberrations introduced by finite data resolution, instrument effects, noise and artefacts of data reduction. More recently, a method to determine the structure of organic compounds from the PDF by skipping indexing has been proposed (Schlesinger *et al.*, 2021 ▸), but it relies on extensive user control and is actually limited to rigid organic molecules. On the other hand, deep-learning approaches have been developed to determine the space group (Liu *et al.*, 2019 ▸; Lan *et al.*, 2022 ▸) and extract structural motifs (Anker *et al.*, 2022 ▸) from an experimental PDF; a web server is available to perform these calculations in the cloud (Yang *et al.*, 2021 ▸). These pioneering works demonstrate the high scientific interest in extracting as much information as possible from PDF profiles.

In this work, we make a step forward in this direction, as we propose a method to extract the crystal cell parameters directly from a PDF profile. The underlying idea is that peaks corresponding to lattice translations are present in the PDF profile even in the absence of long-range order. Thus, the crystal cell parameters could be, in principle, retrieved also in cases where indexing is hampered by low crystallinity or limited crystallite sizes. For example, our proposed approach would be valuable when investigating nanocrystals prepared by colloidal methods, for which the relatively large sizes (∼50–200 Å) and high crystallinity would allow us to define a proper unit cell. As these synthetic methods are highly tunable and can easily give access to metastable phases, it is not uncommon to obtain materials for which no corresponding bulk structure is known. Therefore, *ab initio* crystal structure solution has recently become a priority in the field of nanocrystals, further motivated by the steady advancements in the number of elements and complexity of materials investigated in colloidal form. One major limitation, however, is that powder diffraction profiles collected on nanomaterials suffer from peak broadening, peak overlap and weak signal. In these conditions, many steps of the structure solution process are hampered: in particular, this leads to the failure of the diffraction pattern indexing, which is the first, fundamental step, preparatory to intensity extraction and phasing. This was recently demonstrated in the work by Toso *et al.* (2020 ▸, 2022 ▸), where the structures of two lead sulfohalides, Pb_4_S_3_Cl_2_ and Pb_3_S_2_Cl_2_, had to be solved by a combination of single-nanocrystal electron diffraction for pattern indexing and powder X-ray diffraction for intensity extraction. Here, a PDF would be an ideal X-ray based alternative, as it would allow us to extract the unit-cell parameters from direct space by dealing with interatomic distances. Indeed, given the local character of the PDF, the cell parameters might be derived even for lattices comprising a few unit cells, thus providing valuable information to assist the indexing of difficult powder diffraction patterns, and even opening us up to the more ambitious possibility of an *ab initio* structure solution performed completely in direct space. Motivated by these perspectives, here we propose a two-stage procedure, where the properties of the crystal lattice are determined by machine learning applied to the PDF profile and the crystal cell parameters are extracted using an approach based on vector superposition algebra combined with multivariate analysis.

## 2. Methods

**Figure 1:**
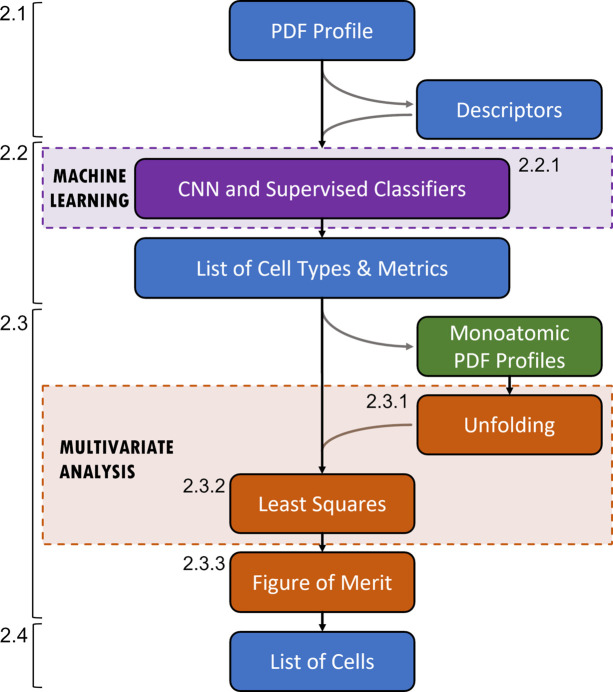
Outline of the steps involved in the procedure to extract cell parameters from a PDF profile. Dashed arrows indicate paths executed depending on the cell type and metric considered. Steps related to machine learning are shown in violet; those related to multivariate analysis are shown in brown. The step in green refers to pre-determined calculations, which are executed when setting up the procedure and are independently on the input PDF profile. The section number where the steps are described are given.

The main steps of the procedure to extract cell parameters from a PDF profile are outlined in Fig. 1 ▸ and explained in the following subsections.

### 2.1. Input data

The input data for the whole procedure is an individual PDF profile. It is used to feed both into the machine-learning algorithms to produce predictions about cell type and metric (Section 2.2) and into multivariate analysis procedures to estimate the crystal cell parameters given the cell type or metric (Section 2.3).

For classification purposes, using the whole PDF profile maximizes the amount of information given to classifier, but it is not necessarily the best choice and an alternative strategy consists of extracting and selecting some characteristics of the PDF profile that could describe it more effectively. To this aim, two different methods have been explored: the recurrence quantitative analysis (RQA) (Marwan & Kurths, 2002 ▸) and the wavelet analysis (Larson, 2007 ▸) (see Section S1 of the supporting information for further details).

In RQA, the descriptors are extracted from the PDF starting from the assumption that the PDF can be seen as the output of a nonlinear dynamic system. Passing through the generation of the Recurrence Plot (a matrix of recurrences of the dynamic system), some characteristics can be extracted such as the intrinsic system dimension, the recurrence of a status in the phase space, the degree of disorder in describing such recurrences and so on. More details are given by Marwan *et al.* (2002 ▸). In the wavelet analysis, instead, the coefficients are a concise descriptor of the PDF seen as a time series. Differently from a Fourier analysis, where only the peak height and width are important (each peak represents a sinusoid), the wavelet coefficients have the property to also catch the location of the peaks through the use of the scale term. In both tested methods, the underlined hypothesis is that a reduced set of descriptors is sufficient to train a classifier since only the relevant information from the PDF is held, discarding any that is not relevant. This hypothesis is often justified by the high sampling usually adopted to generate PDF profiles.

Interestingly and differently from the wavelet coefficients, the RQA descriptors have a physical meaning, so they could be tuned according to specific classification needs of the PDF profiles, such as classifying only subgroups of the cell types. In principle, different sets of RQA descriptors can be generated by changing the inner parameters used to obtain them. An extensive analysis of more RQA descriptors may be a matter of future research.

#### 2.1.1. Training data

The machine-learning tools have been trained on PDF profiles calculated from randomly sampled crystal structures contained in the Crystallography Open Database (COD; Gražulis *et al.*, 2009 ▸). In order to avoid possible bias in the machine-learning session due to uneven population of the different lattice systems, we have fixed the maximum number of entries for each cell type (7000). This number has not been reached for the cubic lattice, where only 4000 entries have been found in the COD. The calculation of the PDF profile from the CIF was accomplished by a Python script that makes use of the *Diffpy-CMI* libraries (Juhás *et al.*, 2015 ▸). PDF profiles have been calculated for interatomic distances between 2 and 40 Å, with a step of 0.01 Å using the following parameters: *Q*_max_ = 30 Å^−1^, *Q*_broad_ = 0.01, *Q*_damp_ = 0.01. The thermal factors originally contained in the CIFs have been read and used for PDF calculation. In case they are absent, isotropic *U* values of 0.01 Å^2^ have been considered for each atom of the compound. This set of parameters ensures a realistic profile generation, which accounts for the thermal motion occurring in real crystals. Given the range of inter­atomic distances considered, crystal structures with a unit cell diagonal higher than 40 Å have been skipped in the COD search. The generation of the PDF profiles for the study of the dependence on crystal size (Section 3.2.2) has been performed by changing the *spdiameter* parameter, which sets the diameter value for the PDF shape-damping function, a spherical-particle PDF correction.

#### 2.1.2. Real data

Experimental PDF profiles of nanocrystal samples have been used to test the crystal cell extraction procedure. Powder diffraction data were collected at the 28ID-2 beamline of the National Synchrotron Light Source (NSLS-II) of Brookhaven National Laboratory with an X-ray energy of 67.17 keV (0.1846 Å) and a 0.5 × 0.5 mm beam size. A Perkin Elmer XRD 1621 digital imaging detector (2048 × 2048 pixels and 200 × 200 µm pixel size) was mounted orthogonal to the beam path about 200 mm downstream from the sample, according to a setup optimized for PDF measurements. Nickel, lanthanum hexaboride (LaB6) and CeO_2_ were measured as standard materials to calibrate the wavelength and the detector geometry, including the sample-to-detector distance. An empty capillary was measured for background estimation. Diffraction images were azimuthally integrated and converted to intensity profiles versus 2ϑ and versus momentum transfer 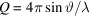 using the *FIT2D* program (Hammersley *et al.*, 1996 ▸). PDF profiles were calculated up to interatomic distances *r* of 40 Å from the *Q* profiles by the program *PDFGetX3* (Juhás *et al.*, 2013 ▸). The parameters for PDF calculation (background subtraction scale factor, minimum and maximum values of *Q*, degree of data-correction polynomial) were optimized on individual PDF profiles, to avoid large termination effects and preserve the signal to noise ratio.

The measured compounds are listed in Table S1 of the supporting information, together with a snapshot of the measured PDF profile. They include:

(i) Orthorhombic [BiSCl, BiSBr (Quarta *et al.*, 2022 ▸)] and trigonal [Bi_13_S_18_Br_2_ (Quarta *et al.*, 2023 ▸)] bis­muth chalcohalides and rhombohedric caesium lead halide [Cs_4_PbBr_6_ (Baranov *et al.*, 2020 ▸)], all characterized by a high crystallinity, since their PDF profiles have relevant peaks up to 35 Å.

(ii) Orthorhombic lead chalcohalides [Pb_4_S_3_I_2_, Pb_4_S_3_Br_2_ (Toso *et al.*, 2022 ▸)] having lower crystallinity, since their PDF profiles have broader peaks up to 30 and 25 Å for Pb_4_S_3_Br_2_ and Pb_4_S_3_I_2_, respectively.

(iii) Tetragonal methyl­ammonium (MA) lead iodide hybrid perovskites obtained by different synthetic routes, which resulted in variations of the relative amount of tetragonal MAPbI_3_ and intermediate PbI_2_–MAI–DMSO (di­methyl sulfoxide) crystal phases (Colella *et al.*, 2018 ▸; Caliandro *et al.*, 2019 ▸).

(iv) Hexagonal tungsten oxide (WO_3_), whose PDF has been measured with a similar experimental setup at the X17A beamline of the former National Synchrotron Light Source (NSLS) at Brookhaven National Laboratory, using X-ray radiation with an energy of 66.7 keV (λ = 0.18597 Å) (Caliandro *et al.*, 2016 ▸).

The *Q*_max_ values determined for the above case studies were between 22 and 30 Å^−1^.

### 2.2. Determination of the cell type and metric

Because a crystalline material repeats identical to itself after any translation corresponding to one of its lattice vectors, any PDF profile must always include a set of peaks found at interatomic distances corresponding to lattice translations. This subset of PDF peaks can be thought of as related to a hypothetical crystal phase whose unit cell consists of a single atom located at the origin (referred to hereafter as a monoatomic unit cell). Besides these, a much larger number of peaks descending from interatomic distances not attributable to lattice translations populates the PDF profile, often overlapping with those corresponding to the monoatomic unit cell distances. The challenging task of recognizing the Bravais lattice from the set of lattice translation distances contained in a PDF profile is attempted here using artificial intelligence. We have used various machine-learning methods for classification of PDF profiles that are described in the following subsection. In the actual implementation, we only take into account primitive cells, so only 7 of the 14 possible Bravais lattices. The Bravais lattices considered are reported in Table 1 ▸, together with the corresponding cell metric and free cell parameters. Two tests were conceived for artificial intelligence: one constituted by the seven lattice systems (Test1), the other by the three cell metric classes (Test2).

#### 2.2.1. Machine-learning methods for classification of PDF profiles

The PDF profile or descriptors extracted from it are used to predict the cell type and metric of the crystalline material without any other prior information. To this aim, different classifiers have been tested, since the classification efficiency depends on both the input data and the algorithm used for classification, and cannot be predicted in advance.

In the first instance, we used a one-dimensional convolutional neural network (CNN) applied to the entire PDF pattern as a one-dimensional input picture. In fact, the main feature of CNNs is the ability to autonomously extract the peculiar features that can lead to the most efficient classification from the provided images. The CNN architecture more suited to process PDF profiles, found after extensive testing, is described in Section S2 of the supporting information.

Then we adopted a set of classifiers implemented in the Python libraries *scikit-learn* (Pedregosa *et al.*, 2011 ▸) and *XGBoost* (Chen & Guestrin, 2016 ▸). Specifically, we used a Dummy classifier (DUM), *i.e.* a classifier that ignores input data, to set a baseline, and then tested the performances of the following classifiers: random forest (RF) (Ho, 1995 ▸), extreme gradient boosting (XGB) (Chen & Guestrin, 2016 ▸), support vector classification (SVC) (Cortes & Vapnik, 1995 ▸) and one based on the *k*-nearest neighbours vote (KNC) (Dasarathy, 1991 ▸). These classifiers have been selected after a preliminary screening among available classifiers and used in their standard configuration, except for KNC, for which the number of neighbours to use has been changed from the default value of 5 to 1, as a result of an optimization targeted to PDF profiles.

In the comparative analysis we did not use CNNs for wavelet coefficients and RQA descriptor data because, by their nature, CNNs are useful for images, or for one-dimensional systems that have a spatial/temporal structure in which the convolution procedure makes sense. This is not the case for tabular data, where column order is not relevant. On the other hand, SVC was not applied to whole PDF profile data, since these classifiers are not suitable to treat a number of descriptors as large as the number of points describing a PDF profile (more than 3000).

All the classifiers have been validated by applying a mean over a 5-repeated 10-fold cross validation. A post-prediction check of global feature extraction has been carried out using the Shapley additive explanations (SHAP) method (Lundberg & Lee, 2017 ▸), which is a game theoretical approach used to explain the output of any machine-learning model, and it is able to give both a global and a local explanation of each feature contribution to the classification.

### 2.3. Extraction of cell parameters

The estimation of the crystal cell parameters given the cell type or metric is performed by multivariate methods implemented in the computer program *RootProf* (Caliandro & Belviso, 2014 ▸). The input PDF profile undergoes a pre-processing step, where the intensity values are rescaled to the interval [0, 1] and the sensitive nonlinear iterative peak (SNIP) algorithm (Morháč *et al.*, 1997 ▸) is applied with a very narrow clipping window (ten data points). The rescaling makes the profile independent of the scattering power of the sample and allows the application of the SNIP algorithm, which requires profiles with positive values, while the SNIP algorithm highlights the PDF features making the positive peaks sharper and resetting the negative ones. Note that this type of pre-processing is not compatible with PDF determined by neutron diffraction, where negative peaks can arise from elements with neutron structure factors of the opposite sign. Thus, neutron PDF cannot be processed by our approach.

**Figure 2:**
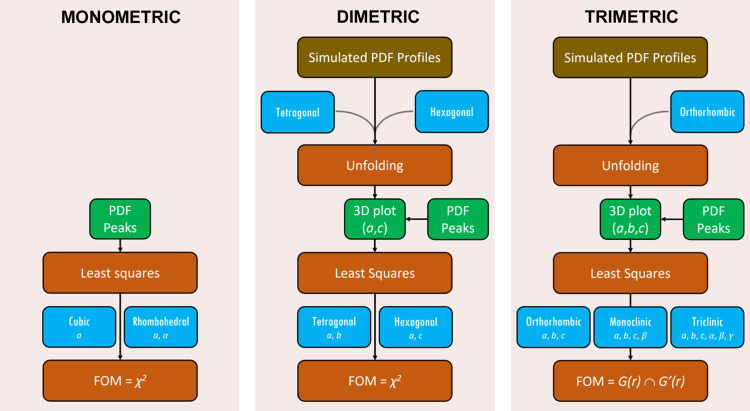
Schematic of the steps for the crystal cell determination from a PDF profile.

The steps involved in the extraction of cell parameters are outlined in Fig. 2 ▸ and explained in the following subsections. The complexity of the procedure increases going from monometric to dimetric and trimetric cells, due to the increasing number of free cell parameters.

#### 2.3.1. The unfolding step

In this step the pre-processed input PDF profile is unfolded with respect to a base set of PDF profiles calculated from geometrically plausible monoatomic unit cells generated for each cell type. The sampling intensity of each unit-cell parameter was determined to ensure a similar number of generated PDF profiles in each lattice system, *i.e.* about 5000. As a result, cell length parameters were generated with a step of 0.4 Å for dimetric cells and 1.7 Å for trimetric cells.

According to the unfolding procedure (Jandel *et al.*, 2004 ▸), the *m* pre-processed monoatomic unit cell profiles are collected in the *m*x*N* response matrix *h*(*i*,*j*), where *N* is the number of data points of the PDF profiles. The weights *w_i_* related to each monoatomic unit cell profile *i* are then calculated by decomposing the input pre-processed PDF profile 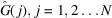 , to the base of monoatomic unit cell profiles *i* = 1, 2… *m*, according to the following equation,

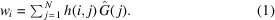

The calculated weights, which can be seen in terms of quantitative analysis as weight fractions of the *i*th monoatomic unit cell profile in the PDF profile 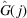 , are then plotted as a function of the corresponding values of the free cell parameters, thus obtaining bidimensional plots in the case of dimetric cells and tridimensional plots in the case of trimetric cells. A peak search procedure applied to these plots supplies the list of cell candidates, which is further checked against the list of peaks extracted from the input pre-processed PDF profile, *i.e.* the coordinates of the peaks found in 2D or 3D plots should separately match the position of at least one peak of the PDF profile by at least 1 Å.

The unfolding procedure is not activated in the case of monometric cells, as unidimensional plots of the unfolding weights are less informative than the list of peaks derived directly from the PDF profile. In addition, in the case of monoclinic or triclinic lattices, the unfolding procedure is executed as it would be for the orthorhombic lattice, because sampling free unit-cell angles would require working with a very large number of monoatomic unit-cell profiles and with plots of dimensions higher than three. Thus, we prefer to maintain a good sampling of the cell length parameters by fixing the angles to 90° as in the orthorhombic case in the unfolding step, and then try to determine the true values of the cell angles through the subsequent least squares step.

#### 2.3.2. The least squares step

In this step, the input pre-processed PDF profile is fitted by a synthetic PDF profile constituted by a set of Gaussians, each one centred at an interatomic distance between a unit-cell node from the origin. These distances are determined by the formula (Giacovazzo, 2006 ▸)

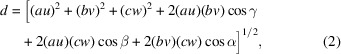

where *a*, *b*, *c*, α, β and γ are the crystal cell parameters and *u*, *v* and *w* are the indices of the unit-cell node considered. These indices can be also negative, as for non-orthogonal cells, the mixed terms in equation (2) have different values depending on the verse in which the unit-cell nodes are taken, and vary in the following ranges,

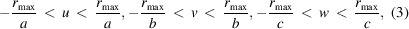

so that the *d* values calculated are always lower than *r*_max_ = 40 Å, *i.e.* the maximum interatomic distance covered by the input PDF profile. The standard deviation of the Gaussian function has been set to a fixed value of 0.1, but may possibly be related to the width of the PDF peaks in the input PDF profile. The fitting function, calculated for each interatomic distance *r* as the maximum among the above set of Gaussians in that point, has the free cell parameters and a normalization constant (Norm) as free fitting parameters. As in standard least squares procedures, the fit is driven by the minimization of the χ^2^ function calculated between the input and the synthetic PDF profiles.

#### 2.3.3. Figures of merit

The ordering of cell candidates optimized by the least squares step follows different criteria depending on the cell metric. For monometric and dimetric cells the χ^2^ function calculated in the least squares step is sufficiently reliable, so it is used to sort the list of cell candidates in increasing order. For trimetric cells, the χ^2^ function does not give a sufficient discrimination of good solutions, so that a figure of merit defined as the intersection between the input pre-processed PDF profile 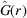 and the synthetic one resulting after least squares optimization *G*′(*r*) is used:

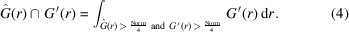

The rationale behind this formula is the following: the number of lattice translation distances is much smaller than that of the distances between atoms made non-equivalent by simple translations. As a consequence, the number of peaks in *G*′(*r*) is much smaller than that in 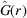 . At higher symmetry (monometric and dimetric cases) a direct comparison between 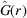 and *G*′(*r*) is still possible, given the small number of PDF peaks. At lower symmetry (trimetric case) the larger number of different interatomic distances worsens the overlap among PDF peaks, making a direct comparison between 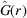 and *G*′(*r*) through the χ^2^ function no longer reliable. The figure of merit of equation (4) is contributed by the peaks of *G*′(*r*) that effectively intersect some of the 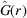 peaks, since only *G*′(*r*) appears as integrand, but it is extended to the region of intersection between the two functions. The threshold value to define the intersection region depends on the normalization constant (Norm) determined in the least squares step.

### 2.4. Output data

The cell extraction procedure generates a list of candidate solutions sorted by the figure of merits described in Section 2.3.3. As the criterion to decide if the true solution has been found within this list, we adopted two different conditions on cell length and angle parameters:

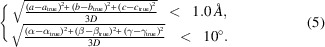

In equation (5) the cell lengths and angles are expressed in Ångstroms and degrees, respectively. The subscript true refers to the true cell parameters of the PDF profile, *i.e.* those reported in the CIF used to calculate it in the case of training data or determined experimentally in the case of real data. The parameter *D* represents the dimension of the problem, *i.e.* *D* = 1 for monometric cells, *D* = 2 for dimetric cells and *D* = 3 for trimetric cells. It has been introduced to account for the increasing difficulty in extracting multi-dimensional information from a unidimensional profile, even considering the sampling intensity of the free cell parameters in the unfolding step. The procedure has been validated by monitoring the first occurrence of a true solution within the list of candidate solutions.

## 3. Results

### 3.1. Cell type and metric determination

**Figure 3:**
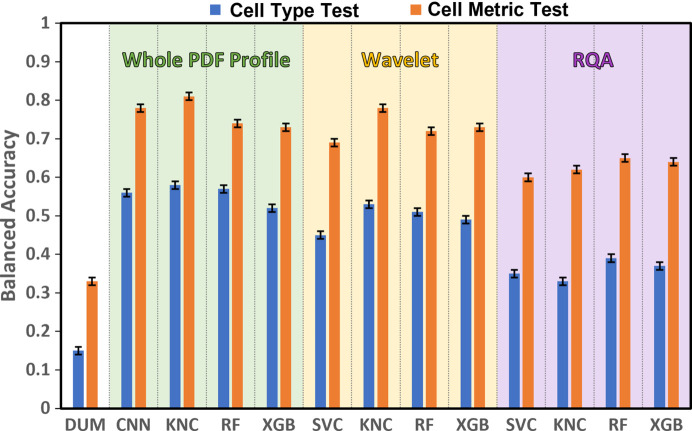
Balanced accuracy in determining the cell type (Test1) and metric (Test2) of different classifiers applied to different descriptors of PDF profiles, *i.e.* whole PDF profile, wavelet coefficients and recurrence quantitative analysis (RQA) descriptors. The balanced accuracy of a dummy classifier (DUM) is shown for comparison. PDF profiles calculated from a fraction of structural models contained in the COD have been used (training PDF profiles).

A benchmark analysis was performed by considering three formats of input data, *i.e.* the whole PDF profile, wavelet coefficients, RQA descriptors and a number of different classifiers. The main results are reported in Fig. 3 ▸ (Section S4 of the supporting information) and summarized in the following:

(i) The trends of the performance in Test1 and Test2 are similar and the balanced accuracy values for Test2 are systematically higher than those of Test1, as expected based on the number of categories in the two tests.

(ii) A hierarchy (whole PDF profile) > (wavelet coefficients) > (RQA descriptors) is followed concerning the type of input data, thus suggesting that using the whole PDF profile is a better strategy than extracting a number of features from it.

(iii) The best classifier is KNC for whole PDF profiles and wavelet coefficients and RF for RQA descriptors. The same results hold for Test1 and Test2.

(iv) The higher values of balanced accuracy are 0.58 ± 0.01 for Test1 and 0.81 ± 0.01 for Test2, attained by the KNC classifier applied to whole PDF profiles.

#### 3.1.1. Classification based on whole PDF profiles

**Figure 4:**
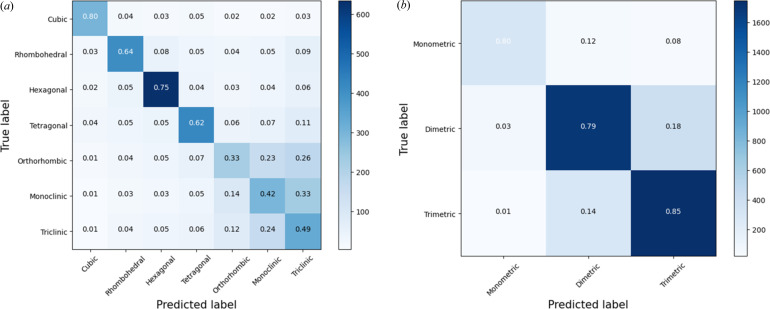
Confusion matrix for (*a*) Test1 and (*b*) Test 2 of the KNC classifier applied to training PDF profiles. Normalized values are shown within the matrix, with boxes coloured based on the number of entries in each box, according to the scale bar on the right.

The normalized confusion matrices for Test1 and Test2 obtained by the KNC classifier on whole PDF profiles are shown in Fig. 4 ▸, those obtained by CNN, RF and XGB classifiers are shown in Figs. S3, S4 and S5, respectively.

From Fig. 4 ▸ it can be noted that the major ambiguities arise among cell types related to trimetric cells. In fact, ortho­rhombic, monoclinic and triclinic cells have probabilities of mutual wrong predictions ranging from 0.12 to 0.33, due to the difficulty to assess deviations of cell angles from 90°. However, these lattices form a well separated cluster, and the corresponding trimetric class has the highest accuracy value in Test2 (0.85). The best classification is obtained by the cubic lattice (0.80). These results justify the assignment of the rhombo­hedral lattice to dimetric cells rather than monometric ones.

**Figure 5:**
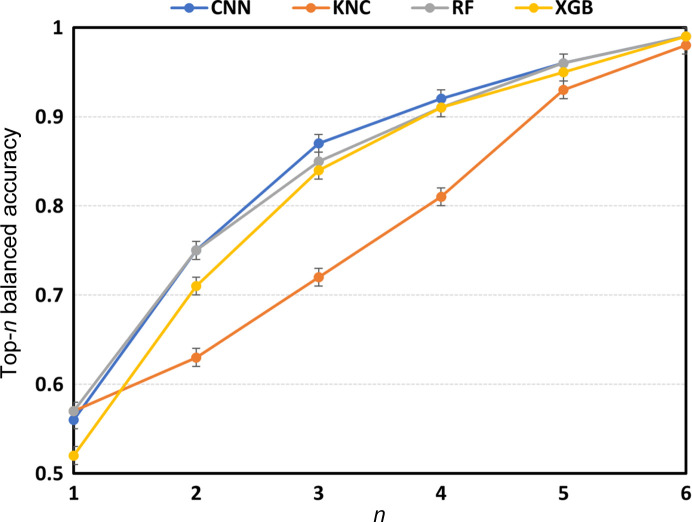
Balanced accuracy in determining the cell type from training PDF profiles by the classifiers shown in the legend when top-*n* predictions are considered.

The analysis of the top-*n* predictions for Test1 (Fig. 4 ▸) highlights that the best classifier (KNC), although having the best top-1 performance, has a slowest growth of accuracy as a function of the number of predictions considered. CNN has instead the higher cumulative accuracy, *i.e.* subtended area under the curve of Fig. 5 ▸. CNN, RF and XGB classifiers reaches 99% accuracy when six predictions are considered.

#### 3.1.2. Classification based on RQA descriptors

**Figure 6:**
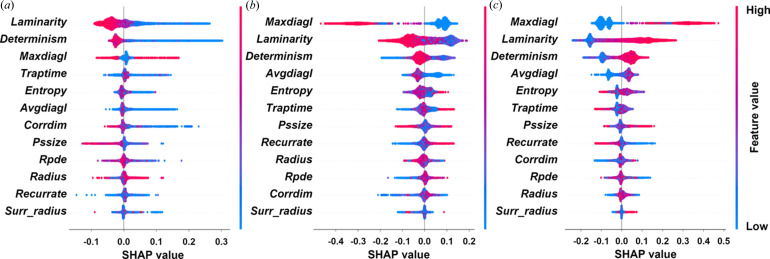
SHAP swarm plots for the (*a*) monometric, (*b*) dimetric and (*c*) trimetric cells, applied to the RF classification of the RQA descriptors of PDF profiles. In these plots each point is a Shapley value for a feature and an instance. The position on the *y* axis is determined by the feature and on the *x* axis by the Shapley value, which represents the impact on the model output. The colour represents the value of the feature from low to high. Overlapping points are jittered in the *y* axis direction, so we get a sense of the distribution of the Shapley values per feature. The features are ordered according to their importance.

The advantage of classification by RQA descriptors is that their physical meaning can be used to understand which characteristic of the PDF profile mostly influences the classification. As an example, the results of the SHAP analysis applied to Test2 considering the RF classifier, which is the best performing in the case of RQA descriptors, are shown in Fig. 6 ▸.

It can be seen that, for the monometric class, *laminarity* and *determinism* are the most important features, whose low values have a great impact on the model output, whereas for the dimetric and trimetric classes the most important feature is *maxdiagl*, whose high values have a high impact on the model output for the trimetric class, but a lower one for the dimetric class. All these recurrence properties relate to the evolution of the unknown dynamic system underlying the PDF (seen as a time series) and its predictability. To this extent, *maxdiagl* can be interpreted as a sort of maximum prediction length in the PDF evolution, *determinism* represents a sort of global predictability of the ‘series’ and *laminarity* represents the occurrence of laminar states in the phase space (Marwan *et al.*, 2002 ▸). These types of plots can be potentially used to find relationships between the considered class (cell type or cell metric) and specific physical properties of the PDF profile, as captured by one of the RQA descriptors.

### 3.2. Crystal cell determination

**Figure 7:**
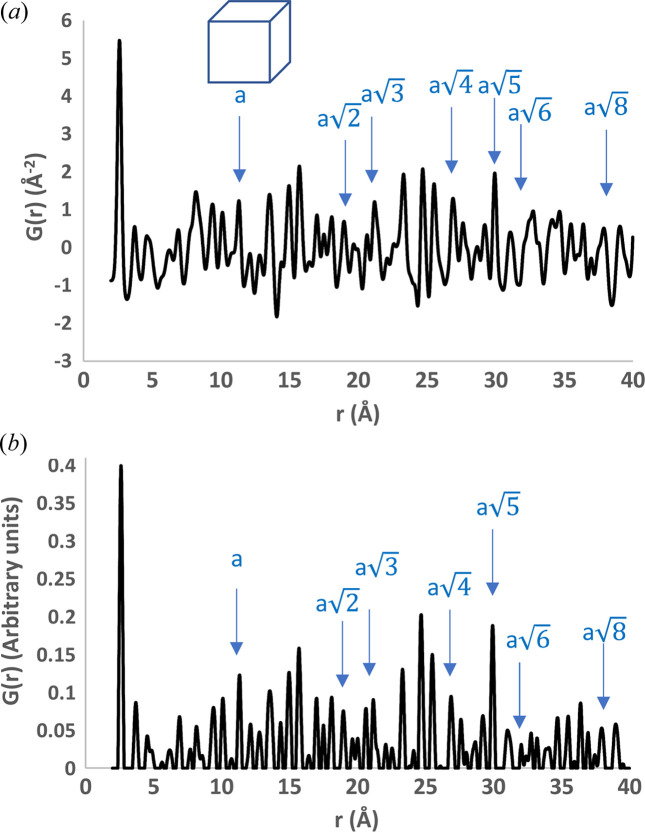
PDF profile calculated from the cubic crystal structure Cu_2_W_6_Br_14_ (Ihmaine *et al.*, 1996 ▸), which has a cubic unit cell with *a* = 13.39 Å, (*a*) before and (*b*) after pre-processing. Arrows indicate the position of the expected peaks due to lattice translations.

To get an idea of the problem to be tackled, the expected interatomic distances due to lattice translations, as determined by applying equation (2), are shown in Fig. 7 ▸ with arrows and listed in Table S2, together with the position of the nearest PDF peak. Note that PDF peaks are generally shifted with respect to their expected position, with deviations up to 0.2 Å. This is due to series termination errors caused by lack of experimental data, which can introduce artificial peaks and oscillations to the data; peak broadening due to atomic thermal motion, which can exacerbate the overlapping of peaks; and superposition with interatomic vectors not related to lattice translations. The rationale of our approach is to overcome these difficulties by performing a consistent search of all the peaks expected for a given crystal cell, so that the effect of peak displacement is reduced by considering all the peaks simultaneously. Though this seems straightforward for the cubic lattice, it becomes challenging when the number of free cell parameters increases.

The effect of pre-processing on the PDF profile is shown in Fig. 7 ▸(*b*): all the peaks become positive and sharper and their overlap is reduced. The positive values can be attributed to the rescaling, while the application of the SNIP algorithm with a small window is responsible for the changes in shape and relative height of the PDF peaks, although their position is only slightly affected.

#### 3.2.1. Results on training data

The procedure to extract the crystal cell parameters has been applied on 1000 training PDF profiles for each lattice system listed in Table 1 ▸, randomly chosen from those used to train and test the machine-learning session. Results obtained by processing the PDF profile shown in Fig. 7 ▸ are detailed in Section S5 of the supporting information.

The CPU time needed for each PDF profile depends on the number of free cell parameters, being on average 2 min for monometric cells, 10–15 min for dimetric cells and from 20 to 180 min for trimetric cells. In the latter case, the free angular cell parameters considerably complicate the cost function hypersurface explored in the least squares procedure, lengthening the processing time (see Section S6 of the supporting information for further details).

**Figure 8:**
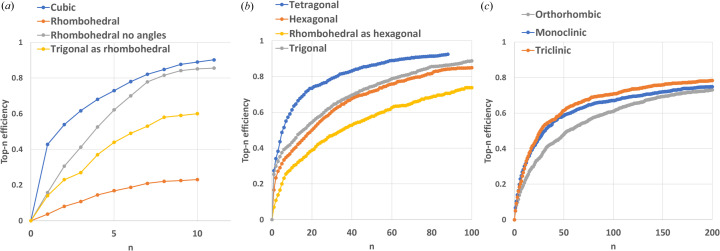
Top-*n* efficiency of the cell parameter extraction procedure, measured as cumulative probability (percentage) of find a good solution, according to the validation criterion of equation (5), in the first *n* solutions, as a function of the rank of the solution. Curves are shown separately for (*a*) monometric, (*b*) dimetric and (*c*) trimetric cells.

The top-*n* efficiency curves determined by applying the validation criterion (5) are shown in Fig. 8 ▸, and their main values are reported in Table S4.

The cell parameter extraction procedure shows very high efficiency for the simplest cubic lattice, with a probability of 43% to find a good solution in the first ranked candidate (top-1 efficiency) and of 90% to find a good solution in the first 11 ranked candidates. A similar efficiency is shown for the rhombohedral lattice only if angle determinations are not checked [‘rhombohedral no angles’ points in Fig. 8 ▸(*a*)]. When instead the threshold on angles is applied in equation (5), the overall efficiency drops from 86 to 23%, thus confirming the difficulty in determining the cell axis directions from a PDF profile.

The dimetric case is characterized by top-1 and top-10 efficiencies of about 20 and 40%, respectively, with a systematically higher efficiency for tetragonal lattices. PDF profiles of crystal structures with trigonal symmetry and a hexagonal cell are processed considering a hexagonal lattice [‘trigonal’ in Fig. 8 ▸(*b*)]. But they can be also processed considering a rhombohedral lattice [‘trigonal as rhombo­hedral’ in Fig. 8 ▸(*a*)], since a hexagonal cell can always be converted to a rhombohedral one via equation (A1[App appa]). On the other hand, crystal structures with trigonal symmetry and a rhombohedral cell can still be processed considering a hexagonal lattice [‘rhombohedral as hexagonal’ in Fig. 8 ▸(*b*)] using equation (A2[App appa]) to convert the rhombohedral cell to a hexagonal one. Comparing the top-*n* efficiency curves shown in Figs. 8 ▸(*a*) and 8 ▸(*b*) related to rhombohedral PDF profiles, it can be concluded that these profiles are more conveniently processed by the procedure developed for dimetric cells and hexagonal lattices. This is the reason why we considered Test2, mapped considering the rhombohedral and hexagonal settings of the trigonal lattice both included in the dimetric case.

The trimetric case shows the lowest efficiencies (top-1 and top-10 efficiencies of about 5 and 30%, respectively) due to the difficulty in determining three axis lengths from a uni­dimensional profile. From Fig. 8 ▸(*c*) it can be noted that the top-*n* efficiency follows a counterintuitive orthorhombic < monoclinic < triclinic hierarchy. This is due to the ambiguity in the assignment of the cell length parameters. In fact, the set of interatomic distances generated by equation (2) is invariant under a permutation of the *a*, *b* and *c* parameters of the orthorhombic cell and of the *a* and *c* parameters of the monoclinic cell. Thus, the least squares procedure, based on equation (2), can produce equivalently cells with these cell parameters permuted, which are however discarded by the validation procedure on the basis of criterion (5). An opposite hierarchy and higher top-*n* efficiencies are obtained if criterion (5) is applied by allowing permutations of cell axis lengths (Fig. S7).

An interesting aspect of the cell parameter extraction procedure developed is that cell axes length predictions are mainly determined by the cell metric, rather than the cell type. In fact, in most cases, reliable values of cell axes lengths can still be achieved if calculations are performed using wrong assignments of the cell type, provided the cell metric is correct (see Section S7.1 of the supporting information for further details). This makes Test2, which has a higher accuracy than Test1, a fundamental source of information to drive the cell parameter extraction process.

#### 3.2.2. Dependence on crystal size, thermal motion and data resolution

**Figure 9:**
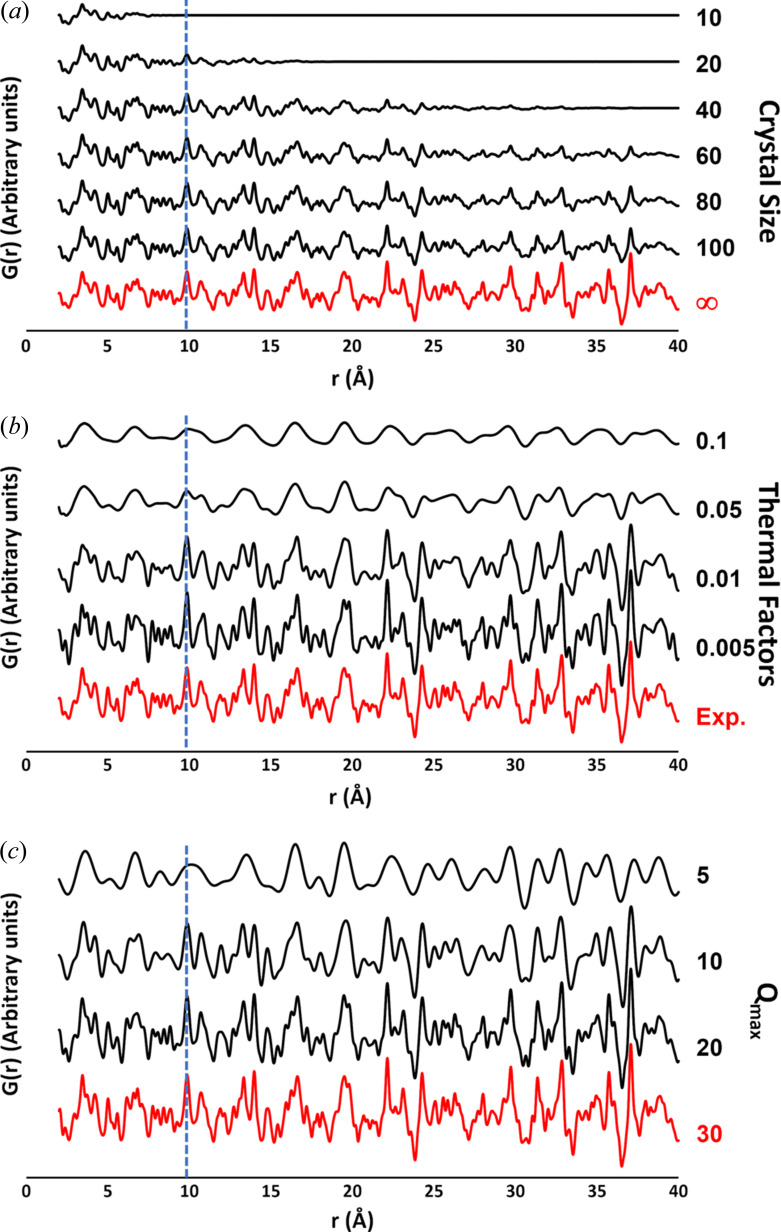
PDF profiles calculated from the cubic langbeinite K_2_Mg_2_O_12_S_3_ (Gajda *et al.*, 2022 ▸) by varying the (*a*) crystal size, (*b*) thermal factors and (*c*) maximum momentum transfer. Numbers on the right refer to: the particle diameter (Å), assuming a spherical shape of the crystal (*a*), the value of the isotropic thermal parameter *U*_iso_ (Å^2^), assumed to be equal for all the atoms (*b*), and the *Q*_max_ value (Å^−1^). The corresponding values used for training data generation are shown in red. A dashed line indicates the peak relevant for the extraction of the cell parameter (*a* = 9.905 Å).

The limits of applicability of the crystal cell determination procedure have been explored by considering PDF profiles calculated from a cubic mineral [langbeinite, K_2_Mg_2_O_12_S_3_ (Gajda *et al.*, 2022 ▸)] at different values of particle diameter [Fig. 9 ▸(*a*)], atomic thermal factor [Fig. 9 ▸(*b*)] and data resolution [Fig. 9 ▸(*c*)]. Profiles reported in Fig. 9 ▸(*a*) exhibit an increasing damping of their features at higher interatomic distances as the particle diameter decreases. The mineral has a crystal cell with *a* = 9.905 Å and the crystal cell extraction procedure finds the first solution at *a* = 9.9 Å for diameter values greater than 50 Å. At 40 Å the right solution is found at the eighth position, with *a* = 9.4 Å, and at 20 Å the closest solution is the fourth, with *a* = 8.9 Å. This instability is determined by the least squares step, where the fit of the damped PDF profile is problematic due to lack of peaks at large *r* values. No solution is found at 10 Å, where even the relevant peak disappears. The amount of thermal motion of individual atoms is another factor that heavily affects PDF profiles, broadening their peaks [Fig. 9 ▸(*b*)]. For *U*_iso_ > 0.01 Å^2^ the large peak overlap erases most of the profile features. The right solution is found at the first position up to *U*_iso_ = 0.05 Å^2^. The decrease in data resolution also manifests itself with a broadening of the peaks accompanied by a loss of information from the PDF profile [Fig. 9 ▸(*c*)]. Here the correct solution is found at the first position even for *Q*_max_ = 5 Å^−1^.

The effects of crystal size, thermal motion and limited data resolution come into play when reaching the nanoscale, prompted by defects, lattice distortions and higher surface area, so that the synthetic PDF profiles generated here are a rough approximation of the experimental ones. Nevertheless, this study shows that a cell-extraction procedure applied in direct space could in principle be successful when the size of the nanocrystal is at least 40 Å, which coincides with the upper limit chosen for analysing the PDF profiles, and in the specific case considered represents a length comprising only four crystal cells.

### 3.3. Results on real data

The cell-extraction procedure calibrated and tested on PDF profiles calculated from known structural models has been applied to experimental PDF profiles. Cell type and metric predictions have been performed in the best conditions, *i.e.* using classifiers trained on whole PDF profiles. The results are summarized in Table 2 ▸ and detailed in Section S8 of the supporting information.

We carried out preliminary tests on the procedure using three calibrants typically used in PDF measurements. They have a cubic cell and exhibit PDF profiles showing high crystallinity, large crystal size and reduced thermal motion (Table S1). The cell parameter is estimated with high precision as the first solution, as the peaks corresponding to the good solution emerge clearly in their PDF profiles. The performance of machine learning in predicting the cell type and metric is instead unsatisfactory, due to the fact that the features of the PDF profiles are substantially different from those for which the procedure has been trained, as can be seen by comparing calibrant profiles with nanocrystal profiles in Table S1.

We then considered the performances on nanocrystal samples, which is the objective of the work. Here, the cell metric is correctly determined by at least two classifiers in their top-1 prediction for all the nanocrystals apart from the methyl­ammonium lead iodide perovskites which, however, have a centred crystal cell, not considered for training in this work. Predictions are more reliable for orthorhombic nanocrystals (BiSCl, BiSBr, Pb_4_S_3_I_2_ and Pb_4_S_3_Br_2_), for which all four classifiers correctly predict a trimetric cell. The cell metrics of trigonal nanocrystals (Bi_13_S_18_Br_2_ and Cs_4_PbBr_6_) are instead correctly predicted by two of the four classifiers used. Cell-type predictions are less accurate, as at most two classifiers produce correct assignments. Note that, for all the samples, compatible predictions of cell metric and cell type are supplied by the same classifier.

Regarding cell parameter estimation, reliable results are provided for nanocrystals with dimetric cells. Cell parameters close to the true ones are found at the first candidate solution for Bi_13_S_18_Br_2_ and at the fourth candidate solution for Cs_4_PbBr_6_. For both these trigonal compounds the cell angles of the rhombohedral cell are predicted with high accuracy, contrary to what has been seen for training PDF profiles, probably due to the fact that their values are close to 90°. Despite the centred cell, not treated in this work, good cell solutions are provided for the three tetragonal lead halide perovskite samples, and their rank increases from 2 to 6 depending on the level of purity of the dominant MAPbI_3_ crystal phase. In this case, the challenge is not in the quality of the PDF profile, but in the presence of a intermediate crystal phase with a weight fraction up to 40%. A good result is also achieved for the hexagonal tungsten oxide nanocrystal, even if its PDF profile was acquired using a less brilliant X-ray beam (NSLS) than that used for the previous samples (NSLS-II).

In the case of trimetric cells, cell parameters close to the true ones are found at the first candidate solution for BiSBr, but only at the 22nd candidate solution for BiSCl, even if the first candidate solution (6.5, 8.6, 5.1 Å) has permuted cell axis lengths. A good solution is instead found in 9th and 15th rank for Pb_4_S_3_Br_2_ and Pb_4_S_3_I_2_, respectively (8th and 4th, respectively, if axis length permutations are considered), but these lead chalcohalides have PDF profiles showing shorter-range order, *i.e.* a rapidly decreasing PDF envelope and larger PDF peaks than those of bis­muth chalcohalides (Table S1).

## 4. Discussion

The major novelty of this work is the procedure to extract cell parameters directly from PDF profiles. The main difficulty encountered is to single out PDF peaks corresponding to interatomic distances due to lattice translations out of the multitude of peaks owing to all the possible interatomic distances. To overcome this problem, an optimized pre-processing of the PDF profile makes its peaks sharper and with minimum overlap. A fitting procedure is then applied to mitigate possible peak shifts and to identify the correct solutions through proper figures of merit. For monometric cells, the fitting procedure can be applied to all the PDF peaks, given their low number due to the high symmetry, whereas for dimetric and trimetric cells it must be coupled with a procedure based on the unfolding algorithm to make a preliminary selection of the PDF peaks to process. Hence, the unfolding procedure discriminates the peaks due to lattice translations based on the comparison with a base set consisting of PDF profiles calculated from hypothetical monoatomic crystal structures and obtained by varying the free cell parameters in a systematic way. It produces a set of candidate solutions searched in 2D (for a dimetric cell) and 3D (for a trimetric cell) space, which are locally refined by the above mentioned fitting procedure.

It is important to underline the following aspects of the implemented procedure:

(i) It has been carefully calibrated, by a realistic choice of the parameters used for PDF profile generation and by balancing the number of PDF profiles among the different cell types.

(ii) It follows different protocols according to the cell metric, to account for the different complexity in the three cases. As a consequence, the results of the procedure do not depend critically on cell-type predictions, while they are mainly affected by cell metric predictions.

(iii) It has been designed to run on a laptop. Besides limiting the CPU time, and thus the number of possible cell solutions to process by the least-squares procedure, this implies a limitation in the memory required by the unfolding step. From this choice follows the undersampling applied in the dimetric and trimetric cases, where cell length parameters have been sampled by a step of 0.4 and 1.7 Å, respectively. It is then clear that with this choice the cell parameters of trimetric cells were difficult to find with a precision lower than 1 Å, which is typically required for crystal structure determination.

Concerning the cell type and metric predictions, the results obtained allow us to clarify that machine learning performs better when applied to whole PDF profiles than to descriptors derived from them. However, descriptors can be useful to create synergy between the lattice identification step and the cell parameter extraction step. For this purpose, procedures such as SHAP analysis help to clarify the role of individual descriptors in the classification. In this perspective: (i) the descriptors obtained by recurrence analysis could be further developed to make explicit their relationship with cell parameters; (ii) CNN, KNC, RF and XGB classifiers could be used in combination within a consensus system, where the same predictions arising from different classifiers are considered more reliable; (iii) greater accuracy could be achieved if cell type and metric predictions are not considered independently, but they are considered more reliable if they are compatible.

We envisage that the procedure could be improved by performing more extensive calculations in the following directions:

(i) Training the cell-type determination procedure on larger datasets of training PDF profiles, enlarging the number of crystal structures considered or the set of parameters used for profile generation.

(ii) Improving the unfolding step in the cell parameter extraction procedure by performing a denser sampling of the cell parameter space in dimetric and especially trimetric cells.

(iii) Performing a search of PDF peaks based on a mathematical model of the PDF profile, thus replacing our empirical pre-processing with algorithms like those developed by Granlund *et al.* (2015 ▸) or Gu *et al.* (2019 ▸).

(iv) Combining the cell parameter extraction procedure on the PDF profile with indexing carried out on the powder diffraction profile measured on the same sample, which could be as effective as it proved to be when direct and reciprocal space operations were combined in the framework of phasing methods.

## 5. Conclusions

A novel method to extract the crystal cell parameters from a PDF profile is reported. It is useful for the cases in which reciprocal-space information is not reliable, such as in materials with limited size, crystallinity or long-range order. In addition, it could complement indexing results that are not conclusive due to experimental problems, such as limited data resolution, preferred orientation effects or high thermal motion. The method has been trained on a large dataset comprising 210 000 PDF profiles calculated from known crystal structures and applied on 13 experimental PDF profiles.

As a first step, the cell type is assessed by using machine learning. Several classifiers have been tested to process PDF profiles, reaching a balanced accuracy of 58% for top-1 estimates, which results in a 81% accuracy for a classification based on the three possible cell metrics (monometric, dimetric and trimetric). An alternative approach based on the use of descriptors extracted from individual PDF profiles considered as time series is less effective, but has the advantage that the descriptors could be linked to specific properties of the PDF profile, which opens to the possibility to apply machine learning in a not-blind mode.

The results of the machine learning feed the second step of the method, where the crystal cell parameters are extracted from the PDF profiles by means of multivariate analysis combined with vector superposition techniques. The overall results on training PDF profiles are very good for monometric cells, where the correct crystal cell parameters are identified in the first ten solutions 90% of the time, and the top-1 solution is correct 43% of the time. For dimetric cells the top-1 solution is correct 20% of the time (40% efficiency for the top-10 solutions), and it decreases to 5% for trimetric cells (30% efficiency for the top-10 solutions). When applied to real data, the cell extraction procedure provides cell parameters compatible with the correct ones in the very first candidate solutions for most of the nanocrystals analysed, even in the presence of a minority crystal phase present with a weight fraction up to 40%.

The method here proposed represents a step towards the model-independent interpretation of PDF data, and paves the way to the development of an *ab initio* crystal structure solution initiated in direct space, where the assessment of the unit cell properties is the first step towards crystal structure determination.

## 6. Related literature

The following references are cited in the supporting information: Tipler (1979 ▸); Kira & Rendell (1992 ▸); Coifman *et al.* (1994 ▸).

## Supplementary Material

Supporting figures and tables. DOI: 10.1107/S2052252523006887/ct5021sup1.pdf

## Figures and Tables

**Table 1:** Subset of Bravais lattices considered in this study The symbol (P) indicates primitive lattices. The crystal cell metric and free parameters are also reported.

Lattice type	Cell metric	Free cell parameters
Cubic (P)	Monometric	*a*
Rhombohedral		*a*, α
Hexagonal	Dimetric	*a*, *c*
Tetragonal (P)		*a*, *c*
Orthorhombic (P)	Trimetric	*a*, *b*, *c*
Monoclinic (P)		*a*, *b*, *c*, β
Triclinic (P)		*a*, *b*, *c*, α, β, γ

**Table 2:** Results of the cell parameter extraction procedure on experimental PDF profiles Only top-1 successful cell type and metric predictions are reported, referred to the following classifiers: CNN, KNC, RF and XGB. Values in bold indicate correct predictions. We also listed the values of the free unit-cell parameters determined by indexing and structural refinement of the X-ray powder diffraction profile (true unit-cell parameters), those estimated by our procedure, determined by selecting the first candidate solution that fulfils conditions reported in equation (5) (estimated unit-cell parameters) and the position of the selected solution in the list of candidate solutions sorted by the figure of merit (order of solution). For compounds with trigonal symmetry (Bi_13_S_18_Br_2_ and Cs_4_PbBr_6_), both the rhombohedral and the hexagonal cell parameters are reported, separated by a slash.

Chemical formula	Cell metric	Cell type	True unit-cell parameters (Å)	Estimated unit-cell parameters (Å)	Order of solution
Calibrants					
Ni	Dim, Trim, Dim, Dim	Tetr, Tric, Hex, Hex	3.6	3.6	1
LaB6	Dim, Dim, Dim, Dim	Tetr, Tetr, Hex, Hex	4.3	4.3	1
CeO_2_	Dim, Dim, Dim, Dim	**Cub**, Hex, Hex, Hex	5.4	5.4	1
Nanocrystals					
BiSCl	**Trim**, **Trim**, **Trim**, **Trim**	**Orth**, Tricl, Hex, **Orth**	7.9 Å, 4.1 Å, 9.2 Å	10.2 Å, 5.1 Å, 8.2 Å	22
BiSBr	**Trim**, Dim, **Trim**, **Trim**	Tricl, Hex, **Orth**, Hex	8.2 Å, 9.9 Å, 4.1 Å	8.2 Å, 7.0 Å, 4.1 Å	1
Bi_13_S_18_Br_2_	Trim, **Dim**, **Dim**, Trim	Tricl, **Hex**, **Hex**, Tricl	9.0 Å, 118° / 15.5 Å, 4.0 Å	9.0 Å, 118° / No solution	1 / –
Pb_4_S_3_I_2_	**Trim**, **Trim**, **Trim**, **Trim**	Tricl, Tricl, **Orth**, Tricl	8.2 Å, 15.6, 8.2 Å	9.7 Å, 14.3 Å, 6.3 Å	15
Pb_4_S_3_Br_2_	**Trim**, **Trim**, **Trim**, **Trim**	Tricl, Tricl, **Orth**, **Orth**	8.2 Å, 14.6 Å, 8.1 Å	6.0 Å, 13.7 Å, 9.7 Å	9
Cs_4_PbBr_6_	Trim, **Dim**, **Dim**, Trim	Tricl, **Rhom**, Hex, Tricl	9.8 Å, 89° / 13.7 Å, 17.3 Å	9.1 Å, 89° / 12.7 Å, 16.1 Å	4 / 20
MAPbI_3_	Trim, Trim, Trim, Trim	Orth, Orth, Orth, Orth	8.9 Å, 12.7 Å	8.9 Å, 11.0 Å	2
MAPbI_3_(0.8) +PbI_2_–MAI–DMSO(0.2)	Trim, Trim, Trim, Trim	Mon, Orth, Orth, Orth	8.9 Å, 12.7 Å	8.9 Å, 13.0 Å	3
MAPbI_3_(0.6) +PbI_2_–MAI–DMSO(0.4)	Trim, Trim, Trim, Trim	Mon, Orth, Mon, Mon	8.9 Å, 12.7 Å	8.9 Å, 13.0 Å	6
WO_3_	Trim, **Dim**, **Dim**, **Dim**	Tricl, **Hex**, **Hex**, **Hex**	7.4 Å, 3.8 Å	7.5 Å, 3.7 Å	2

## Appendix A Conversion formulae between hexagonal and rhombohedral cells

A primitive hexagonal cell can be converted into a triple rhombohedral cell. Let (*a*_H_, *b*_H_, *c*_H_, α_H_, β_H_, γ_H_) be the crystal cell parameters of the hexagonal cell, then the crystal cell parameters of the rhombohedral cell (*a*_R_, *b*_R_, *c*_R_, α_R_, β_R_, γ_R_) can be calculated using the following equations,

which are valid for both obverse and reverse settings (Giacovazzo, 2006 ▸). On the other side, a primitive rhombohedral cell can be converted to a triple hexagonal cell using the following equations,
