# Supplementary material for: Towards the extraction of the crystal cell parameters from pair distribution function profiles
Source: IUCrJ. 2023 Sep 1;10(Pt 5):610–23. doi: 10.1107/S2052252523006887 (PMC10478520; doi:10.1107/S2052252523006887)
Supplement: Supplementary file 1 [file m-10-00610-sup1.pdf]

IUCrJ

Volume 10 (2023)

Supporting information for article:

Towards the extraction of the crystal cell parameters from pair distribution function profiles

Pietro Guccione, Domenico Diacono, Stefano Toso and Rocco Caliandro

S1. Descriptors of the PDF profile

A crystalline structure is intrinsically a three-dimensional system, while the PDF is a mono-dimensional "view" of such system. It is likely that most of the characteristics of the 3D systems are lost in this view. In the research field of dynamic systems, scholars are used to deal with 1D measures out of a multi-dimensional (and, often, nonlinear) dynamic systems. However, dynamic systems are time-evolving while PDF are distribution of distances. Recurrence, which is at the basis of the methodology used to "interpret" the PDF as a time series, is a fundamental mechanism inside many aspects of reality (Tipler, 1979). Extending the fundamental Poincarè Recurrence Theorem (and the derived theory, on which the parameters used in the following ground on) from dynamical systems in time to "dynamical" systems in space is not formally correct, but it is a not too exotic leap. So, and with some degree of caution, we *assimilate the PDF profile to the time series produced by an unknown dynamic system*. Based on this assumption, the height, width and position of the PDF peaks are expression of intrinsic characteristics of the system we want to unveil. Methods to analyse such dynamic systems are based mainly on the goal we pursue. In our case, we aim at identifying common properties from the analysis of the PDF profile.

S1.1. Selection of Wavelet coefficients

Classification is based on the idea that a set of descriptors are sufficient to determine an intrinsic property of a sample, said class. The availability of a large set of descriptors may hinder the classification, since: (i) some descriptors can be redundant, i.e. they can be achieved (usually by linear combination) from the existing ones; (ii) some descriptors can be inversely related to class, inducing misclassification; (iii) some descriptors can be totally uncorrelated to the class and so generate useless noise in the classification stage. For this reason, the descriptors extracted from the Wavelet analysis (whose number can be very high) have been reduced to a reasonable number.

The selection of the best descriptors has been done using the Relief algorithm (Kira *et al.*, 1992). Relief calculates a feature score for each descriptor which is applied to rank and select the top scoring features for a strict selection. The Wavelet coefficients have been extracted using the Bump base function (Coifman *et al.*, 1994) and a frequency interval [1,4] achieving a total of 210 coefficients. In Figure S2, the relative importance of the Wavelet coefficients after the ranking given by the Relief algorithm is reported.

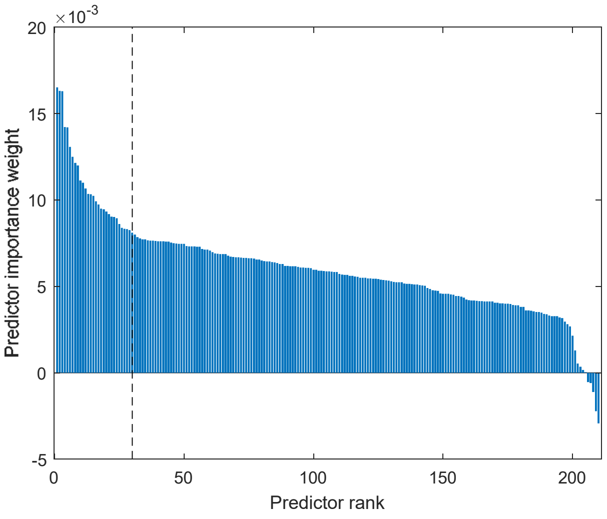

Figure S1 Relief algorithm applied to the set of Wavelet coefficients extracted from the PDF profile seen as a time series. The cell type classification has been set as goal for the Relief algorithm. The black dotted vertical line indicates the cut-off value that selects coefficients used to make classification (the first 30).

Instead RQA descriptors have been held without any ranking, since only 12 descriptors have been extracted. RQA is, in fact, highly time consuming and, for this reason, a specific set of inner parameters have been fixed, after the tuning using a reduced set of data. No selection has been performed when considering whole PDF profiles.

References:

Kira, K. and Rendell, L. (1992). The Feature Selection Problem: Traditional Methods and a New Algorithm. AAAI-92 Proceedings.

Coifman R. R., Meyer Y., Quake S., Wickerhauser M. V., Signal processing and compression with wavelet packets. Chapter 285. Part of the NATO ASI Series book series (ASIC, volume 442). 1994.

Tipler, F. General relativity, thermodynamics, and the Poincaré cycle. (1979). *Nature* **280**, 203–205.

S2. Description of the Convolutional Neural Network optimized to process PDF profiles

There is no predefined way to build a CNN that is efficient for the problem addressed: it is a matter of programmer experience, and trying various configurations of the hyperparameters to find the most suitable set for the particular classification task that is being made. The final architecture of the CNN optimized to process PDF profiles is given in Figure S1.

The features extraction section is constituted by 3 convolutional blocks, each formed by a Conv1D layer followed by BatchNormalization, Activation, and Dropout layers. The number of Conv1D filters is 80 in each Conv1D layer. The kernel size starts with 100 and is divided by 2 in the second block and by 4 in the third one.

Other parameters are padding = “same” and activation function = “relu”. The Dropout rate is 0.25 in each block. The Flatten layer is followed by the classification section, constituted by 2 densely connected blocks, each formed by a Dense layer followed by BatchNormalization, Activation, and Dropout layers. The number of neurons used in the Dense layer is 700 and 70.

The last block is followed by the output layer formed by 7 units (one for each crystal class), with “softmax” activation function, to ensure that the sum of the seven output neuron values is always equal to 1. We used tha Adam optimizer, with a learning rate determined by an exponential decay scheduler.

The Early Stopping approach, with patience = 20, was adopted to minimize the network overfitting, i.e., training was stopped at the point when performance on a validation dataset starts to degrade, and at the end of the fitting phase, the best CNN weights were restored.

The 1D - CNN was originally designed for classification according to the cell type (Test1). By only changing the output layer to three neurons, the network was retrained for classification of PDFs according to the cell metric (Test2).

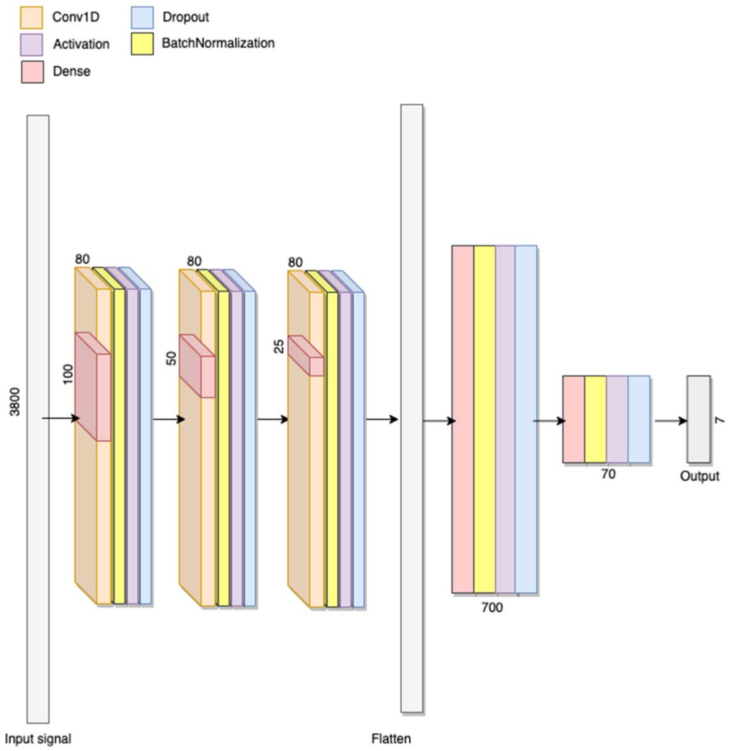

Figure S2 Architecture of the convolutional neural network optimized to process PDF profiles.

S3. Nanocrystal samples used to test the cell parameter extraction procedure

Table S1 Chemical formula, symmetry and experimental PDF profiles of the real structures used to test the cell parameters extraction procedure, together with the reference to recent publications where the PDF data have been used for structural characterization.

Chemical formula	Space group	PDF profile	Reference
Ni	F m$\bar{3}$m	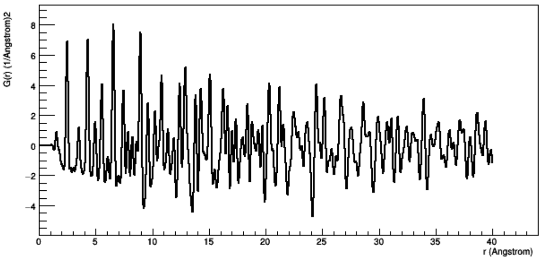	Calibrant
LaB6	P m$\bar{3}$m	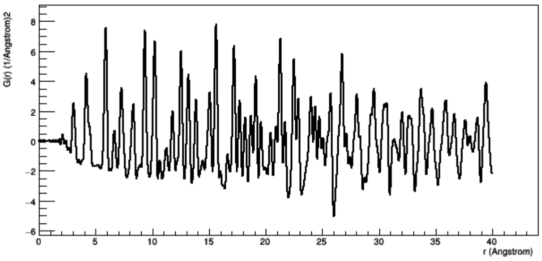	Calibrant
CeO_2	F m$\bar{3}$m	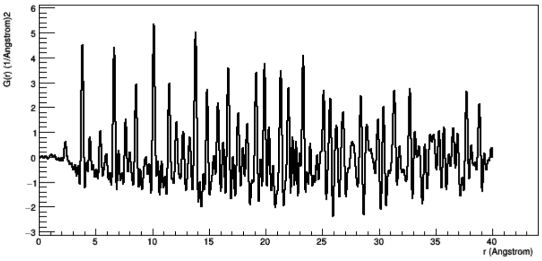	Calibrant
BiSCl	P nma	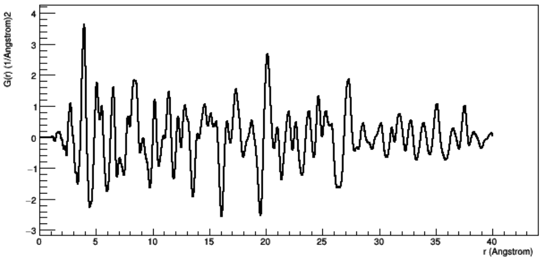	Quarta *et al.*, (2022)

BiSBr	P nam	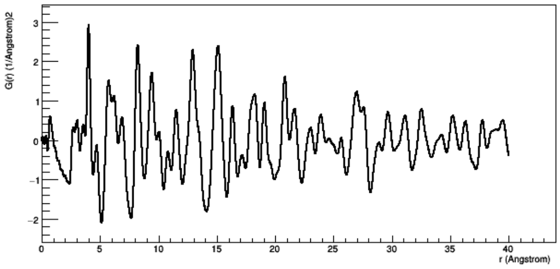	Quarta *et al.*, (2022)
$Bi_{13}S_{18}Br_2$	P 3	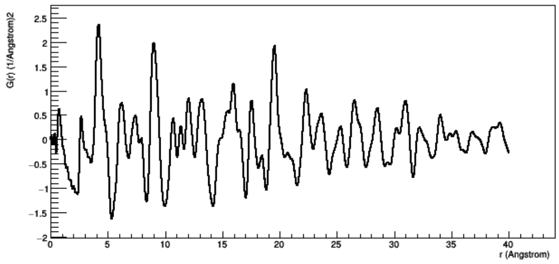	Quarta *et al.*, (2023)
$Pb_4S_3I_2$	P nma	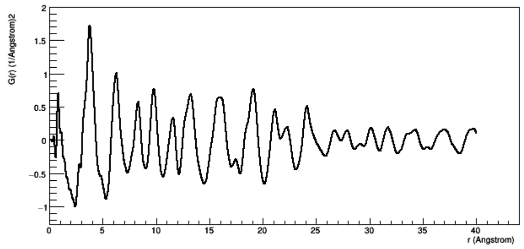	Toso *et al.* (2022)
$Pb_4S_3Br_2$	P nma	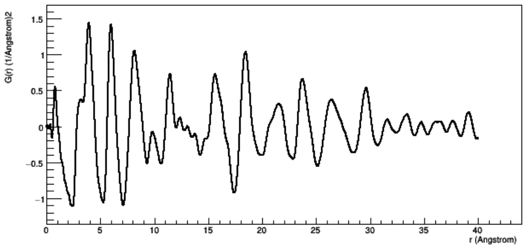	Toso *et al.* (2022)
Cs_4PbBr_6	R -3 c	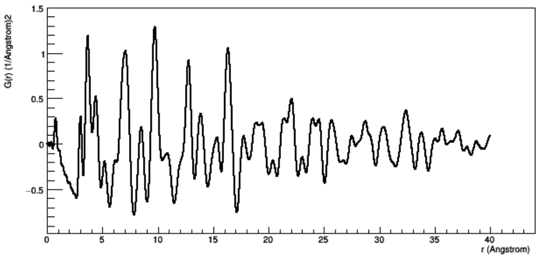	Baranov *et al.*, (2020)

$MAPbI_3$	I 4/mcm	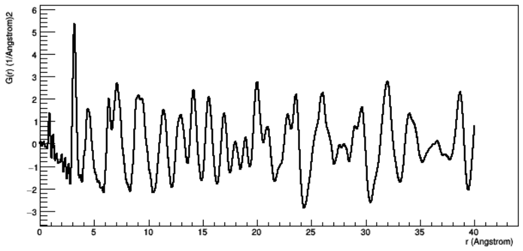	Colella *et al.*, (2018)
$MAPbI_3$ (0.6)+ PbI_2–MAI–DMSO (0.4)*	I 4/mcm	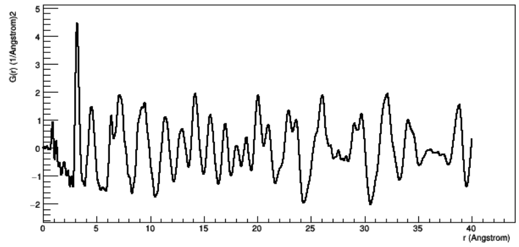	Caliandro *et al.*, (2019)
$MAPbI_3$ (0.8)+ PbI_2–MAI–DMSO (0.2)**	I 4/mcm	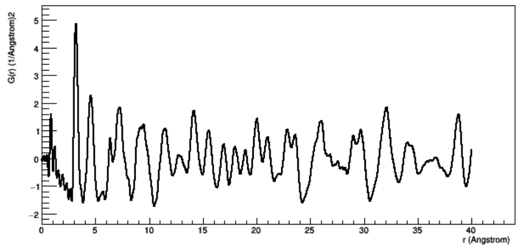	Caliandro *et al.*, (2019)
WO_3	P 6/mmm	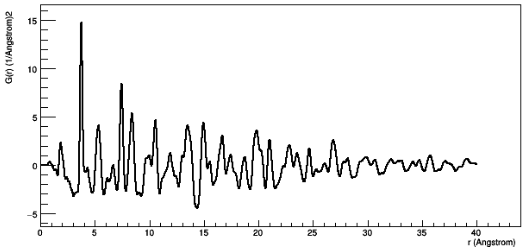	Caliandro *et al.*, (2016)

* It corresponds to the sample Pero2 in Caliandro *et al.*, (2019).

** It corresponds to the sample Pero1 in Caliandro *et al.*, (2019).

S4. Results of machine learning on training PDF profiles

The input PDF profiles have been assigned to the cell types (Test1) based on the space group of the original compound from which the PDF profile has been calculated, following to the standard relationship between crystal system and Bravais lattices. In the case of the trigonal system, compounds determined in the rhombohedral primitive cell have been assigned to the rhombohedral lattice, while those determined in the P cell have been assigned to the hexagonal lattice. The assignments related to the cell metric (Test2) are instead the following: Cubic system → Monometric cell, Tetragonal, Hexagonal and Trigonal systems → Dimetric cell, Orthorhombic, Monoclinic and Triclinic systems → Trimetric cell. Thus, the trigonal system in its rhombohedral and hexagonal settings is completely included into the dimetric case and space groups with rhombohedral system are not included in the monometric class. This choice derives from results obtained by machine learning for Test2 (paragraph 3.1.1) and by the cell parameters extraction procedure (paragraph 3.2.2).

It should be mentioned that, despite Test1 and Test2 are carried out independently, they are in fact interconnected, and Test2 predictions could be derived from those of Test1 by using algebraic formulas. Results obtained in this way (not shown) does not differ significantly from those obtained by carrying out the two tests independently.

The figure of merit chosen to compare the performance of the various classifiers is the Balanced Accuracy, defined as the arithmetic mean of two commonly used metrics: sensitivity (also known as true positive rate) and specificity (also known as true negative rate). It is calculated as the average of the recall obtained on each class, where recall is the fraction of relevant instances that were retrieved, achieved by the ratio between each element on the diagonal of the confusion matrix divided by the sum of the corresponding row.

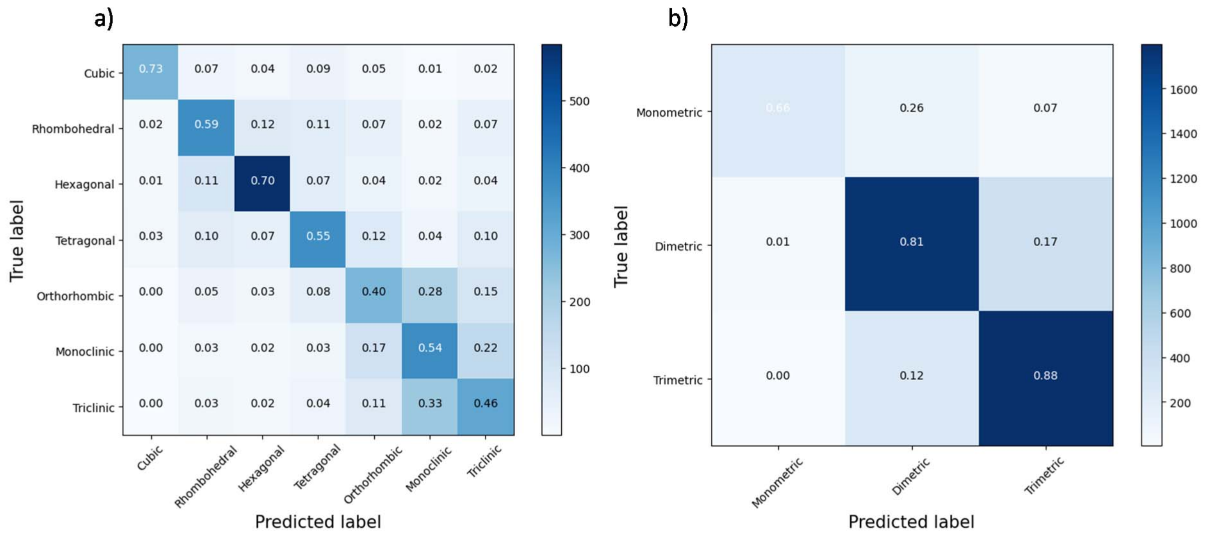

Figure S3 Confusion matrix for Test1 (a) and Test2 (b) of the CNN classifier applied to training PDF profiles. Normalized values are shown within the matrix, with boxes coloured based on the number of entries in each box, according to the scale bar on the right

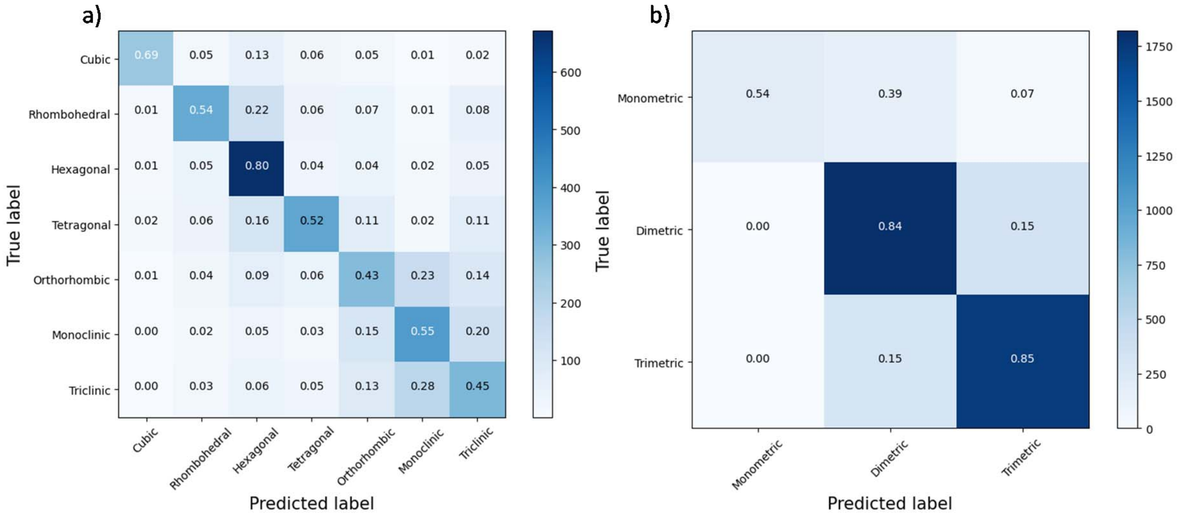

Figure S4 Confusion matrix for Test1 (a) and Test2 (b) of the RF classifier applied to training PDF profiles. Normalized values are shown within the matrix, with boxes coloured based on the number of entries in each box, according to the scale bar on the right.

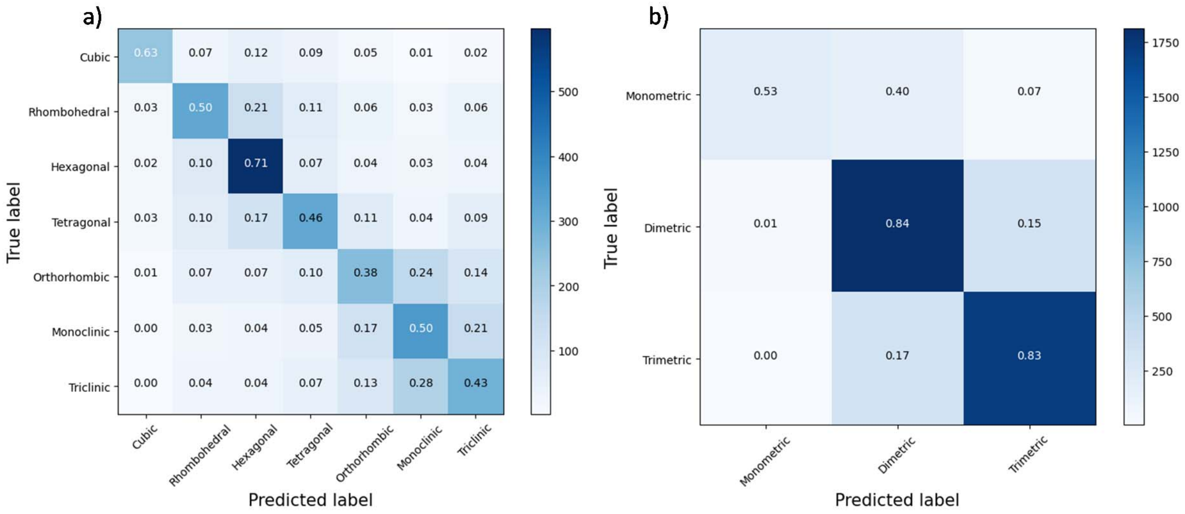

Figure S5 Confusion matrix for Test1 (a) and Test2 (b) of the XGB classifier applied to training PDF profiles. Normalized values are shown within the matrix, with boxes coloured based on the number of entries in each box, according to the scale bar on the right.

S5. Case study: the cubic crystal structure $Cu_2W_6Br_{14}$

Table S2 List of lengths of vectors due to lattice translation expected in the range 2-40 Å spanned by the PDF profile for a cubic cell with cell parameter *a*=13.39 Å.

Cell vector length	Drawing	Expected interatomic distance (Å)	Position of the nearest PDF peak (Å)
a		13.39	13.55
$a\sqrt{2}$	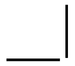	18.93	18.95
$a\sqrt{3}$	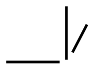	23.19	23.30
$a\sqrt{4}$		26.78	26.90
$a\sqrt{5}$	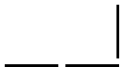	29.93	29.95
$a\sqrt{6}$	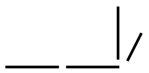	32.79	32.75
$a\sqrt{8}$	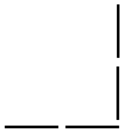	37.86	37.95

The application of the cell parameter extraction procedure to the PDF profile shown in Figure 7 produced the following list of candidate solutions:

```
List of best solutions for crystal system Cubic:
Order    FOM        a     b     c       alpha beta gamma
 1      8.186      5.00 5.00 5.00       90.0 90.0 90.0
 2      8.832      7.07 7.07 7.07       90.0 90.0 90.0
 3      9.296      8.28 8.28 8.28       90.0 90.0 90.0
 4      9.386      14.96 14.96 14.96    90.0 90.0 90.0
 5      9.445      18.06 18.06 18.06    90.0 90.0 90.0
 6      9.480      10.10 10.10 10.10    90.0 90.0 90.0
 7      9.533      11.39 11.39 11.39    90.0 90.0 90.0
 8      9.551      18.99 18.99 18.99    90.0 90.0 90.0
 9      9.597      12.76 12.76 12.76    90.0 90.0 90.0
 10     9.670      15.87 15.87 15.87    90.0 90.0 90.0
 11     9.715      16.98 16.98 16.98    90.0 90.0 90.0
 12     9.716      20.64 20.64 20.64    90.0 90.0 90.0
```

The validation criterion of eq. (5) identified the solution n.9, having cell parameter a=12.76 Å as a good solution, since the its root mean squared error with respect to the true value a_{true}=13.55 Å is 0.63 Å, below the threshold value of 1.0 Å. It is worth noting that:

-- the least squares procedure applied to the selected solution shifted by only 0.04 Å the cell parameter a with respect to its initial value determined by the PDF peak position;

-- solution n.8 has a=18.99 Å, very close to one of the expected cell vector length ($a\sqrt{2}$=18.93 Å) according to Table S2.

S6. Execution times of the procedure for the extraction of the cell parameters from a PDF profile

Table S3 Average execution time for processing a single PDF profile. N_{length} is the number of free cell axis lengths, N_{angle} is the number of free cell angles, N_{trial} is the average number of trials to process by least squares.

Cell type	Average execution time (min)	N_{length}	N_{angle}	N_{trial}
Cubic (P)	2	1	0	10
Trigonal rhombohedral	2	1	1	7
Hexagonal (P)	15	2	0	120
Tetragonal (P)	10	2	0	100
Orthorhombic (P)	20	3	0	500
Monoclinic (P)	120	3	1	500
Triclinic (P)	180	3	3	500

A better agreement with data is reached if the execution time is correlated with the number of free cell parameters so that the cell angles are weighted 5 times more than the cell lengths (Figure S6).

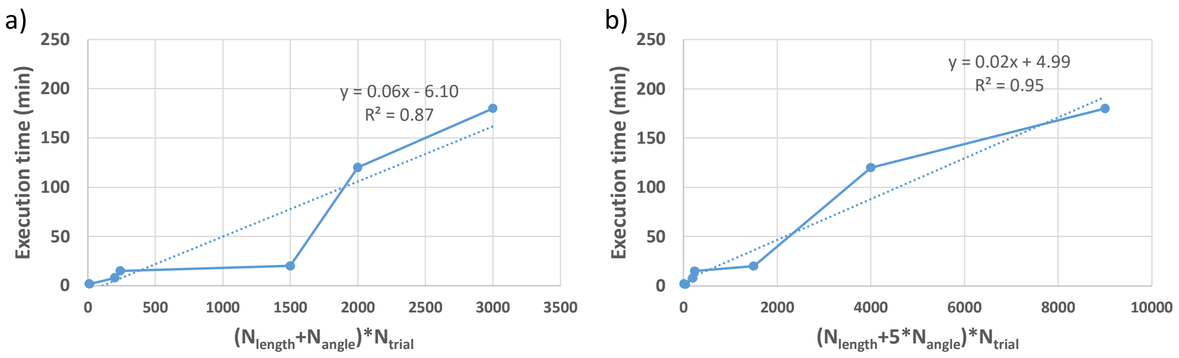

Figure S6 Execution time versus two possible combinations of the parameters. A linear regression line is shown in dashed line, and the corresponding equation and R^2 factor is reported.

S7. Results of the cell parameter extraction procedure on training PDF profiles

Table S4 Efficiency of the cell parameter extraction procedure, measured as probability (in percentage) to find the solution, according to the validation criterion of eq. (5), within the given rank of candidates. “Rhombohedral no angle” reports the results of the validation procedure where the root mean square error on angles is not taken into account. “Rhombohedral as hexagonal” and “Trigonal as hexagonal” report the results of the validation procedure for crystal structures belonging to the trigonal crystal system and described respectively by a rhombohedral and a hexagonal P cell, both processed by the procedure developed for hexagonal lattice.

Cell metric	Cell type	1	<5	<10	<15	<20	Max
Monometric	Cubic	43	73	89			90 (11)
Monometric	Rhombohedral	4	17	23			23 (10)
Monometric	Rhombohedral no angle	16	62	85			86 (11)
Monometric	Trigonal as rhombohedral	14	44	60			60 (10)
Dimetric	Rhombohedral as hexagonal	7	20	29	34	39	80 (139)
Dimetric	Trigonal	25	38	43	49	54	91 (123)
Dimetric	Hexagonal	17	31	38	44	50	87 (127)
Dimetric	Tetragonal	27	49	61	69	74	93 (88)
Trimetric	Orthorhombic	5	14	21	27	31	78 (495)
Trimetric	Monoclinic	7	20	30	39	41	79 (485)
Trimetric	Triclinic	5	18	29	38	44	83 (474)

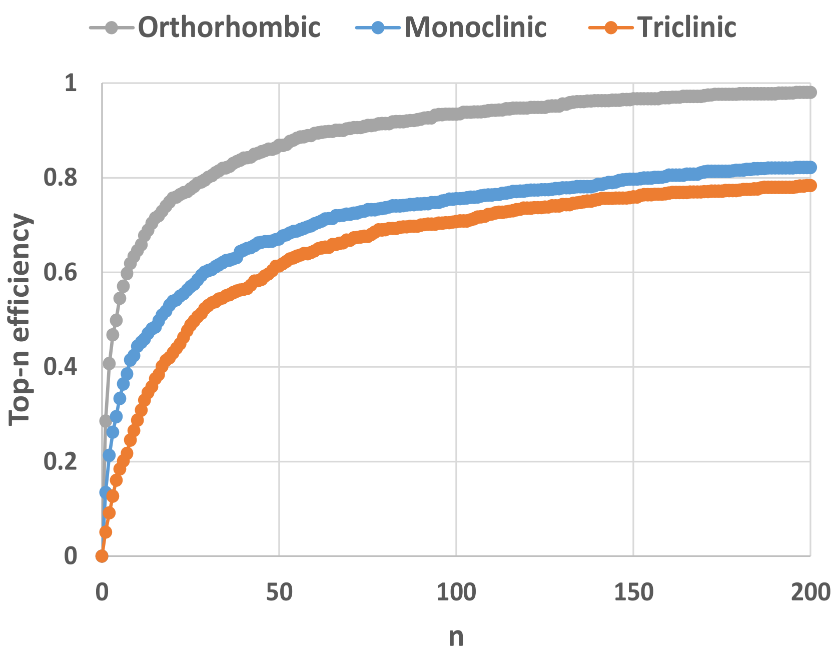

Figure S7 Top-n efficiency of the cell parameter extraction procedure applied to trimetric cells. It has been measured as cumulative probability (percentage) of find a good solution in the first *n* solutions, as a function of the rank of the solution. The validation criterion of eq. (5) has been applied by allowing permutations of *a*, *b*, *c* cell axis lengths for orthorhombic lattice and of *a*, *c* for monoclinic lattice.

S7.1. Results of the cell parameter extraction procedure on training PDF profiles in case of wrong assignment of the cell type within the same cell metric class

The overall efficiencies obtained in these conditions are reported in Table S5, and the top-5 efficiencies are compared with those obtained in case of right assignment of the cell type in Figure S8. A slight decrease of efficiency is observed for the cubic lattice interpreted as rhombohedral and a higher efficiency is obtained by processing rhombohedral lattices as cubic ones, probably because the least-squares procedure is more performant without free angular variables. A more pronounced efficiency reduction is observed for wrong assignments in dimetric and trimetric cells, and a dramatic decrease of efficiency is obtained when orthorhombic cells are misinterpreted as monoclinic, for the same reason mentioned before, and when tetragonal lattices are misinterpreted as hexagonal ones, probably because the unfolding procedure is affected by a poor sampling of the tetragonal parameters by monoatomic hexagonal cells.

Table S5 Efficiency in estimating the length of the cell axes calculated in case of wrong assignments of the cell type. The threshold on angles (eq. 5b) has not been used in this case.

Cell type	Cell type used for calculations	1	<5	<10	<15	<20	Max
Cubic	Rhombohedral	21	60				77 (8)
Rhombohedral	Cubic	21	68	82			82 (10)
Hexagonal	Tetragonal	8	21	33	40	44	71 (80)
Tetragonal	Hexagonal	4	13	21	27	33	68 (122)
Orthorhombic	Monoclinic	0	2	3	7	7	26 (266)
Monoclinic	Orthorhombic	1	9	15	22	24	65 (428)
Triclinic	Orthorhombic	2	8	14	23	29	73 (391)

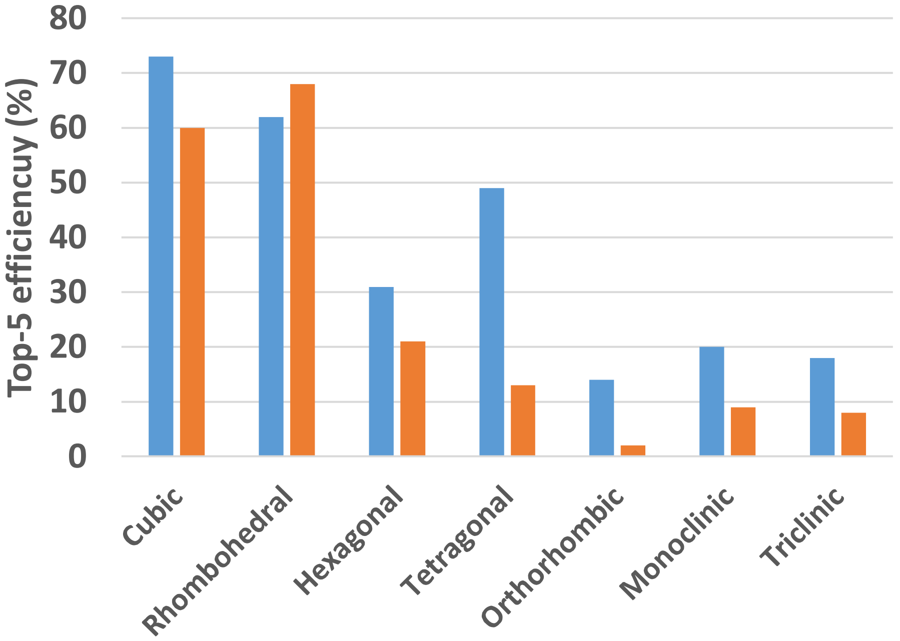

Figure S8 Top-5 efficiency for the estimation of the crystal cell axes lengths (blue bars) compared with the same efficiency obtained by using a wrong assignment of the cell type within the same cell metric (orange bars).

S8. Results of machine learning on experimental PDF profiles

It should be noted that the probability values calculated on the actual profiles are the result of averaging over those calculated during the cross validation procedure.

Table S6 Probability values obtained by the CNN classification based on whole PDF profiles applied to experimental PDF profiles. Shaded cells indicate the true assignments of cell metric and cell type, while values in bold indicate the cases in which the highest probability predicted by the classifier coincide with the true assignment.

Chemical formula	Cell metric			Cell type						
	Mon.	Dim.	Trim.	Cub.	Romb.	Hex.	Tetr.	Orth.	Mono.	Tric.
Ni	0.00	0.99	0.01	0.01	0.30	0.27	0.36	0.03	0.00	0.03
LaB6	0.31	0.69	0.00	0.42	0.03	0.08	0.47	0.00	0.00	0.00
CeO_2	0.34	0.63	0.03	**0.54**	0.10	0.26	0.09	0.01	0.00	0.01
BiSCl	0.00	0.18	**0.82**	0.00	0.02	0.02	0.07	**0.32**	0.28	0.29
BiSBr	0.01	0.13	**0.87**	0.00	0.00	0.02	0.01	0.12	0.41	0.44
$Bi_{13}S_{18}Br_2$	0.01	0.29	0.70	0.01	0.03	0.04	0.07	0.12	0.22	0.51
$Pb_4S_3I_2$	0.04	0.28	**0.68**	0.02	0.06	0.02	0.15	0.12	0.14	0.49
$Pb_4S_3Br_2$	0.05	0.35	**0.61**	0.02	0.03	0.03	0.13	0.11	0.15	0.53
Cs_4PbBr_6	0.01	0.31	0.68	0.00	0.01	0.02	0.02	0.02	0.07	0.87
$MAPbI_3$	0.03	0.23	0.74	0.04	0.08	0.05	0.13	0.26	0.23	0.21
$MAPbI_3$(0.8) +PbI_2–MAI–DMSO(0.2)	0.02	0.17	0.81	0.02	0.04	0.03	0.08	0.24	0.30	0.28
$MAPbI_3$(0.6) +PbI_2–MAI–DMSO(0.4)	0.00	0.10	0.89	0.01	0.01	0.02	0.04	0.24	0.36	0.32
WO_3	0.00	0.24	0.76	0.01	0.02	0.04	0.02	0.04	0.13	0.75

Table S7 Probability values obtained by the KNC classification based on whole PDF profiles applied to experimental PDF profiles. Shaded cells indicate the true assignments of cell metric and cell

type, while values in bold indicate the cases in which the highest probability predicted by the classifier coincide with the true assignment.

Chemical formula	Cell metric			Cell type						
	Mon.	Dim.	Trim.	Cub.	Romb.	Hex.	Tetr.	Orth.	Mono.	Tric.
Ni	0.00	0.00	1.00	0.00	0.00	0.00	0.00	0.00	0.00	1.00
LaB6	0.00	1.00	0.00	0.00	0.00	0.00	1.00	0.00	0.00	0.00
CeO_2	0.00	1.00	0.00	0.00	0.00	0.90	0.10	0.00	0.00	0.00
BiSCl	0.00	0.00	**1.00**	0.00	0.00	0.00	0.00	0.00	0.00	1.00
BiSBr	0.00	0.90	0.10	0.00	0.00	0.90	0.00	0.00	0.02	0.08
$Bi_{13}S_{18}Br_2$	0.00	**0.98**	0.02	0.0	0.00	**0.98**	0.00	0.00	0.00	0.02
$Pb_4S_3I_2$	0.00	0.10	**0.90**	0.00	0.10	0.00	0.00	0.00	0.00	0.90
$Pb_4S_3Br_2$	0.00	0.06	**0.94**	0.00	0.00	0.00	0.06	0.00	0.04	0.90
Cs_4PbBr_6	0.00	**1.00**	0.00	0.00	**1.00**	0.00	0.00	0.00	0.00	0.00
$MAPbI_3$	0.00	0.00	1.00	0.00	0.00	0.00	0.00	0.90	0.06	0.04
$MAPbI_3$(0.8) +PbI_2–MAI–DMSO(0.2)	0.00	0.00	1.00	0.00	0.00	0.00	0.00	0.90	0.02	0.08
$MAPbI_3$(0.6) +PbI_2–MAI–DMSO(0.4)	0.00	0.00	1.00	0.00	0.00	0.00	0.00	1.00	0.00	0.00
WO_3	0.00	1.00	0.00	0.00	0.00	1.00	0.00	0.00	0.00	0.00

Table S8 Probability values obtained by the RF classification based on whole PDF profiles applied to experimental PDF profiles. Shaded cells indicate the true assignments of cell metric and cell type, while values in bold indicate the cases in which the highest probability predicted by the classifier coincide with the true assignment.

Chemical formula	Cell metric			Cell type						
	Mon.	Dim.	Trim.	Cub.	Romb.	Hex.	Tetr.	Orth.	Mono.	Tric.
Ni	0.06	0.66	0.27	0.07	0.20	0.29	0.17	0.11	0.08	0.09

LaB6	0.10	0.60	0.30	0.08	0.19	0.24	0.14	0.16	0.12	0.07
CeO_2	0.08	0.72	0.20	0.07	0.20	0.37	0.17	0.07	0.06	0.05
BiSCl	0.05	0.43	**0.52**	0.07	0.15	0.23	0.20	0.16	0.11	0.09
BiSBr	0.11	0.40	**0.49**	0.11	0.10	0.18	0.14	**0.18**	0.16	0.14
$Bi_{13}S_{18}Br_2$	0.07	**0.50**	0.43	0.08	0.16	**0.20**	0.18	0.15	0.09	0.14
$Pb_4S_3I_2$	0.05	0.44	**0.51**	0.06	0.11	0.19	0.15	**0.20**	0.11	0.18
$Pb_4S_3Br_2$	0.08	0.43	**0.49**	0.06	0.09	0.13	0.19	**0.20**	0.16	0.17
Cs_4PbBr_6	0.07	**0.49**	0.44	0.07	0.17	0.19	0.18	0.15	0.09	0.16
$MAPbI_3$	0.05	0.43	0.51	0.05	0.12	0.18	0.13	0.20	0.16	0.17
$MAPbI_3$(0.8) +PbI_2–MAI–DMSO(0.2)	0.05	0.34	0.61	0.04	0.09	0.14	0.12	0.21	0.20	0.20
$MAPbI_3$(0.6) +PbI_2–MAI–DMSO(0.4)	0.05	0.34	0.61	0.03	0.08	0.13	0.11	0.21	0.24	0.20
WO_3	0.06	**0.51**	0.43	0.06	0.15	**0.25**	0.13	0.12	0.10	0.19

Table S9 Probability values obtained by the XGB classification based on whole PDF profiles applied to experimental PDF profiles. Shaded cells indicate the true assignments of cell metric and cell type, while values in bold indicate the cases in which the highest probability predicted by the classifier coincide with the true assignment.

**Chemical formula**	**Cell metric**			**Cell type**						
	**Mon.**	**Dim.**	**Trim.**	**Cub.**	**Romb.**	**Hex.**	**Tetr.**	**Orth.**	**Mono.**	**Tric.**
Ni	0.03	0.74	0.23	0.04	0.12	0.35	0.18	0.10	0.08	0.13
LaB6	0.11	0.56	0.33	0.09	0.14	0.37	0.10	0.07	0.17	0.06
CeO_2	0.04	0.87	0.09	0.07	0.21	0.47	0.17	0.03	0.02	0.03
BiSCl	0.05	0.19	**0.76**	0.02	0.08	0.11	0.11	**0.39**	0.20	0.09
BiSBr	0.03	0.48	**0.49**	0.03	0.04	0.29	0.11	0.13	0.14	0.26
$Bi_{13}S_{18}Br_2$	0.03	0.47	0.50	0.04	0.10	0.06	0.22	0.20	0.15	0.22
$Pb_4S_3I_2$	0.02	0.35	**0.63**	0.02	0.05	0.08	0.12	0.25	0.05	0.43

$Pb_4S_3Br_2$	0.02	0.17	**0.82**	0.01	0.01	0.02	0.04	**0.44**	0.11	0.37
Cs_4PbBr_6	0.03	0.38	0.59	0.03	0.03	0.04	0.30	0.12	0.07	0.42
$MAPbI_3$	0.01	0.45	0.54	0.02	0.14	0.22	0.14	0.23	0.14	0.11
$MAPbI_3$(0.8) +PbI_2–MAI–DMSO(0.2)	0.01	0.24	0.75	0.02	0.03	0.15	0.15	0.27	0.19	0.19
$MAPbI_3$(0.6) +PbI_2–MAI–DMSO(0.4)	0.01	0.17	0.82	0.01	0.01	0.16	0.09	0.18	0.34	0.22
WO_3	0.02	**0.65**	0.33	0.02	0.07	**0.54**	0.15	0.06	0.04	0.08
